# Supplementary material for: Kinetic analysis of oncolytic OrfV-induced innate and adaptive immune responses in a murine model of late-stage ovarian cancer
Source: Mol Ther Oncolytics. 2023 Nov 10;31:100748. doi: 10.1016/j.omto.2023.100748 (PMC10701082; doi:10.1016/j.omto.2023.100748)
Supplement: Document S1. Figures S1, S2, Tables S1, and S2 [file mmc1.pdf]

OMTO, Volume *31*

Supplemental information

Kinetic analysis of oncolytic OrfV-induced innate and adaptive immune responses in a murine model of late-stage ovarian cancer

Jessica A. Minott, Jacob P. van Vloten, Jake G.E. Yates, Lisa A. Santry, Kathy Matuszewska, Madison Pereira, Melanie M. Goens, Alicia M. Viloria-Petit, Geoffrey A. Wood, Khalil Karimi, James J. Petrik, Byram W. Bridle, and Sarah K. Wootton

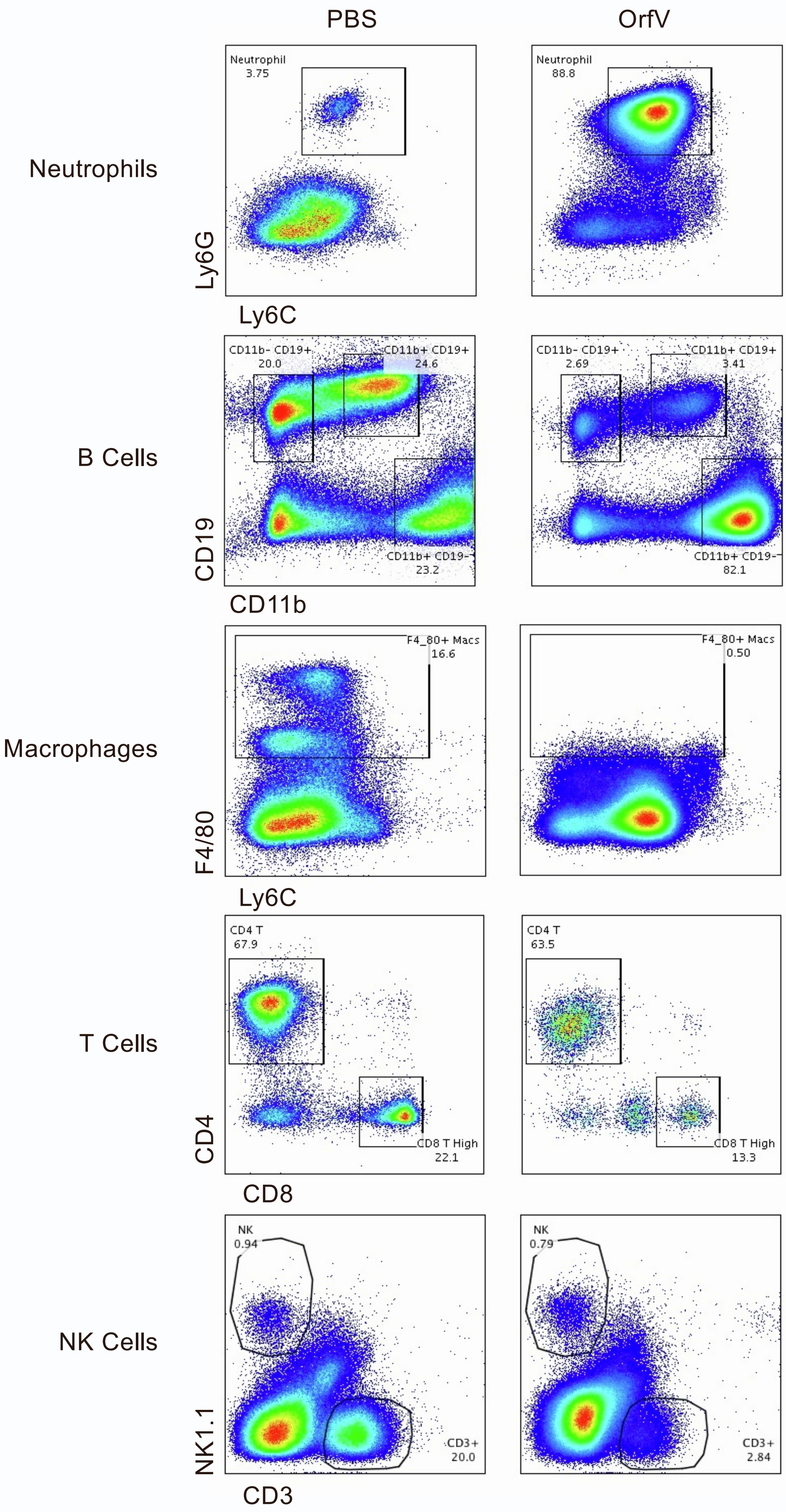

Figure S1: Example of gating used for flow cytometry

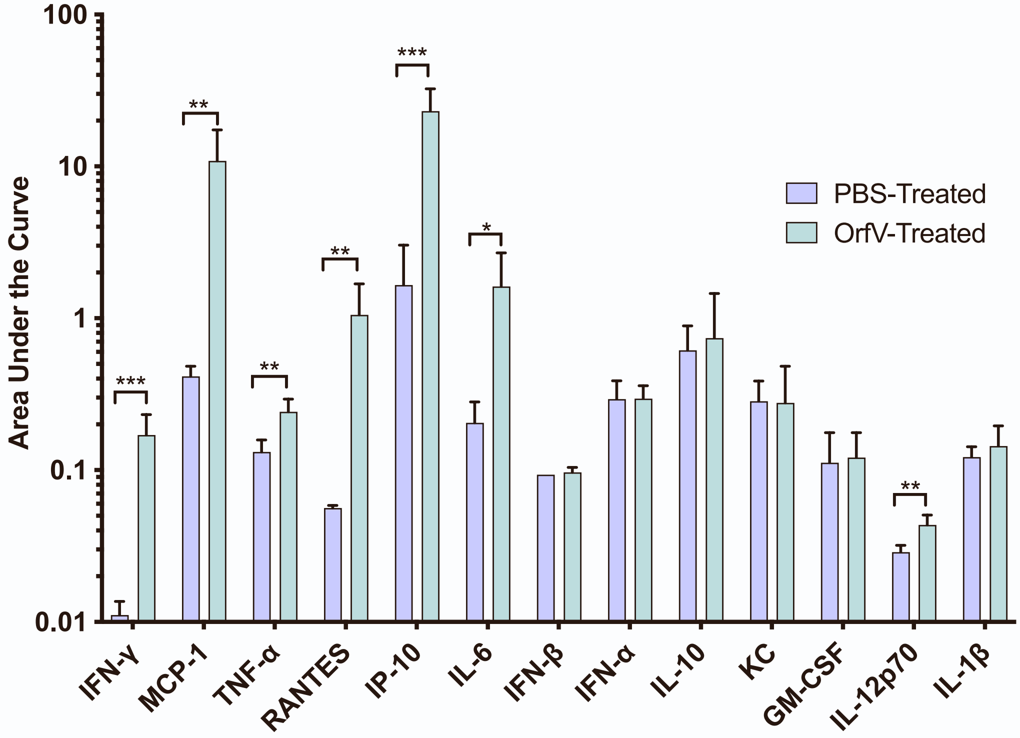

Figure S2. Comparison of the area under the curve for cytokine expression levels in the ascites fluid harvested from OrfV and PBS treated mice and evaluated on days 1, 3, 5, 8, 10, 15, and 21 days post-treatment. Areas under the curve were generated for each individual cytokine and graphically depicted to compare cytokine levels within the peritoneal lavage fluid of OrfV- and PBS-treated mice. P values represent comparison of area under the curve for individual cytokines from PBS or OrfV treated mice using a two-way ANOVA multiple comparisons test. *P<0.05, **P<0.01, ***P<0.001.

Table S1. Markers used for flow cytometry staining to identify specific immunological cell subsets. CD, cluster of differentiation; IFNγ, interferon gamma; MHC- II, major histocompatibility complex molecule II; PD-1, programmed cell death protein 1; PD-L1, Programmed death-ligand 1; TNFα, tumor necrosis factor alpha.

**Leukocyte Subsets**	**Phenotypic Markers**
Neutrophils	CD45.2, Ly6G, Ly6C, CD11b
Macrophages	F4/80, Ly6C, CD45.2, CD11b
NK cells	CD69, NK1.1, CD3ε, CD45.2, CD4, PD-1, PD-L1, CD8α
DCs	CD11b, CD11c, MHC-II, CD8α, CD103, CD40, CD80
cDC1s	CD103, CD11b, CD11c, MHC II, CD8a, CD40, CD80
B cells	CD19, CD45.2
T cells	CD3ε, CD8α, CD4, CD45, IFNγ, TNFα, CD69, PD-1, PD-L1

Table S2. Example staining panel. 7AAD, 7-Aminoactinomycin D; CD, cluster of differentiation; DC, dendritic cell; cDC1, type 1 conventional dendritic cell; FVD, fixable viability dye; IFNγ, interferon gamma; MHC- II, major histocompatibility complex molecule II; NK, natural killer cell; PD-1, programmed cell death protein 1; PD-L1, Programmed death-ligand 1; TNFα, tumor necrosis factor alpha.

	**530 FITC**	**585 PE**	**780B-PECy7**	**670LP PerCP5.5**	**660 APC**	**780R APCCy7**	**450 BV421**	**510 BV510**
Neutrophils, Macrophages and B cells	F4/80 (eBiosciences, Cat#11-4801-85)	Ly6G (BDPharm, Cat#551461)	CD45.2 (BDbio, Cat#560696)	7AAD (Cat#420404)	CD19 (Cat# 152410)	Ly6C (BDbio, Cat#560596)	CD11b (eBioscience, Cat#48-0112-82)	
NK cells	CD69 (Cat#104506)	PD-L1 (Cat# 124308)	CD4 (eBiosciences, Cat#25-0042-82)	PD-1 (Cat#109120)	NK1.1 (Cat#108710)	FVD NIR (Cat#423106)	CD3ε (Cat#100336)	CD8α (Cat#100752)
DCs and cDC1s	CD103	CD80 (eBiosciences, Cat#12-0801-85)	CD11c (Cat#117318)	7AAD	CD40 (eBiosciences, Cat#17-0401-82)	MHC II (eBioscience, Cat#17-5321-82)	CD11b	CD8α
T cells	CD69	TNF-α (eBioscience, Cat#12-7321-82)	CD4	PD-1	IFN-γ (Cat. #505808)	FVD NIR	CD3ε	CD8α
