# Supplementary material for: Kinetic analysis of oncolytic OrfV-induced innate and adaptive immune responses in a murine model of late-stage ovarian cancer
Source: Mol Ther Oncolytics. 2023 Nov 10;31:100748. doi: 10.1016/j.omto.2023.100748 (PMC10701082; doi:10.1016/j.omto.2023.100748)
Supplement: Document S2. Article plus supplemental information [file mmc2.pdf]

Molecular Therapy
Oncolytics
Original Article

Kinetic analysis of oncolytic OrfV-induced innate and adaptive immune responses in a murine model of late-stage ovarian cancer

Jessica A. Minott,[1,3] Jacob P. van Vloten,[1,3] Jake G.E. Yates,[1] Lisa A. Santry,[1] Kathy Matuszewska,[2] Madison Pereira,[2] Melanie M. Goens,[1] Alicia M. Viloria-Petit,[2] Geoffrey A. Wood,[1] Khalil Karimi,[1] James J. Petrik,[2] Byram W. Bridle,[1] and Sarah K. Wootton[1]

[1]Department of Pathobiology, University of Guelph, Guelph, ON N1G 2W1, Canada; [2]Department of Biomedical Sciences, University of Guelph, Guelph, ON N1G 2W1, Canada

Immunotherapies revive host immune responses against tumors by stimulating innate and adaptive immune effector cells with antitumor functions. Thus, detailed studies of immunological cell phenotypes and functions within the tumor microenvironment (TME) following immunotherapy treatments is critical to identifying the determinants of therapeutic success, optimizing treatment regimens, and driving curative outcomes. Oncolytic viruses such as Orf virus (OrfV) are multifunctional biologics that preferentially infect and kill cancer cells while simultaneously causing inflammation that drives anticancer immune responses. Here, we describe the immunological impact of OrfV on the ascites TME in a preclinical model of advanced-stage epithelial ovarian cancer. OrfV promoted the infiltration of several immune effector cells with increased expression of activation markers and effector cytokines into the ascites TME, which correlated with reduced ascites tumor burden and improved survival. The kinetics of the immune response and change in tumor burden following OrfV therapy revealed an optimal re-administration time to sustain antitumor immunity, further extending survival. The data presented highlight the importance of investigating immune response kinetics following immunotherapy and demonstrate that detailed kinetic profiling of immune responses can reveal novel insights into mechanisms of action that can guide the development of more effective therapies.

INTRODUCTION

Epithelial ovarian cancers (EOCs) remain the most lethal among gynecologic malignancies.[1] Ineffective early screening and detection methods often result in late-stage diagnosis characterized by the accumulation of fluid in the abdomen, known as ascites, and the presence of metastatic lesions within the peritoneal cavity. The ascites that develops in EOC patients not only contributes to morbidity and mortality but it also facilitates metastasis and chemoresistance.[2] More than 80% of patients with ovarian cancer experience recurrent disease after chemotherapy and lack other treatment options.[3] Biological therapeutics for EOC represent a promising alternative or complement to conventional chemo- and radiotherapies, including oncolytic virotherapy.

Oncolytic viruses (OVs) preferentially infect and lyse cancer cells while simultaneously stimulating the immune system and inducing antitumor immune responses.[4] Although the mechanism of action of OVs was originally thought to be mediated by viral-induced tumor lysis, there is growing appreciation that OVs function as potent immune stimulatory agents that recondition the tumor microenvironment (TME).[5,6] The ability to stimulate innate and adaptive antitumor immune responses has been identified as a critical component of the therapeutic activity of several different OVs,[5] some of which have demonstrated efficacy even in the absence of lytic activity.[7,8]

One promising OV under recent exploration is the sheep parapoxvirus, Orf virus (OrfV).[9] Although relatively new to the field of cancer therapeutics, OrfV has a number of attributes that make it a promising OV, including potent immunostimulatory properties,[10,11] a lack of preexisting immunity in humans,[12] a large double-stranded DNA genome that can accommodate sizable therapeutic payloads, the ability to re-administer in the presence of humoral immunity,[13] the ability to produce high titers,[14] and demonstrated efficacy in preclinical models of melanoma and colorectal and breast cancers.[15,16] Recently, we have shown OrfV to be an effective monotherapy that extends survival and reduces ascites burden in a murine model of advanced-stage EOC.[17] OrfV intervention activated a robust anticancer immune response that predominantly relied on natural killer (NK) cells, supported by type 1 conventional dendritic cell (cDC1s) and T cell activity.

Received 30 December 2022; accepted 7 November 2023;
https://doi.org/10.1016/j.omto.2023.100748.

[3]These authors contributed equally

Correspondence: Sarah K. Wootton, Department of Pathobiology, University of Guelph, PAHL Room 4836, Guelph, ON N1G 2W1, Canada.
E-mail: kwootton@uoguelph.ca

This is an open access article under the CC BY license (http://creativecommons.org/licenses/by/4.0/).

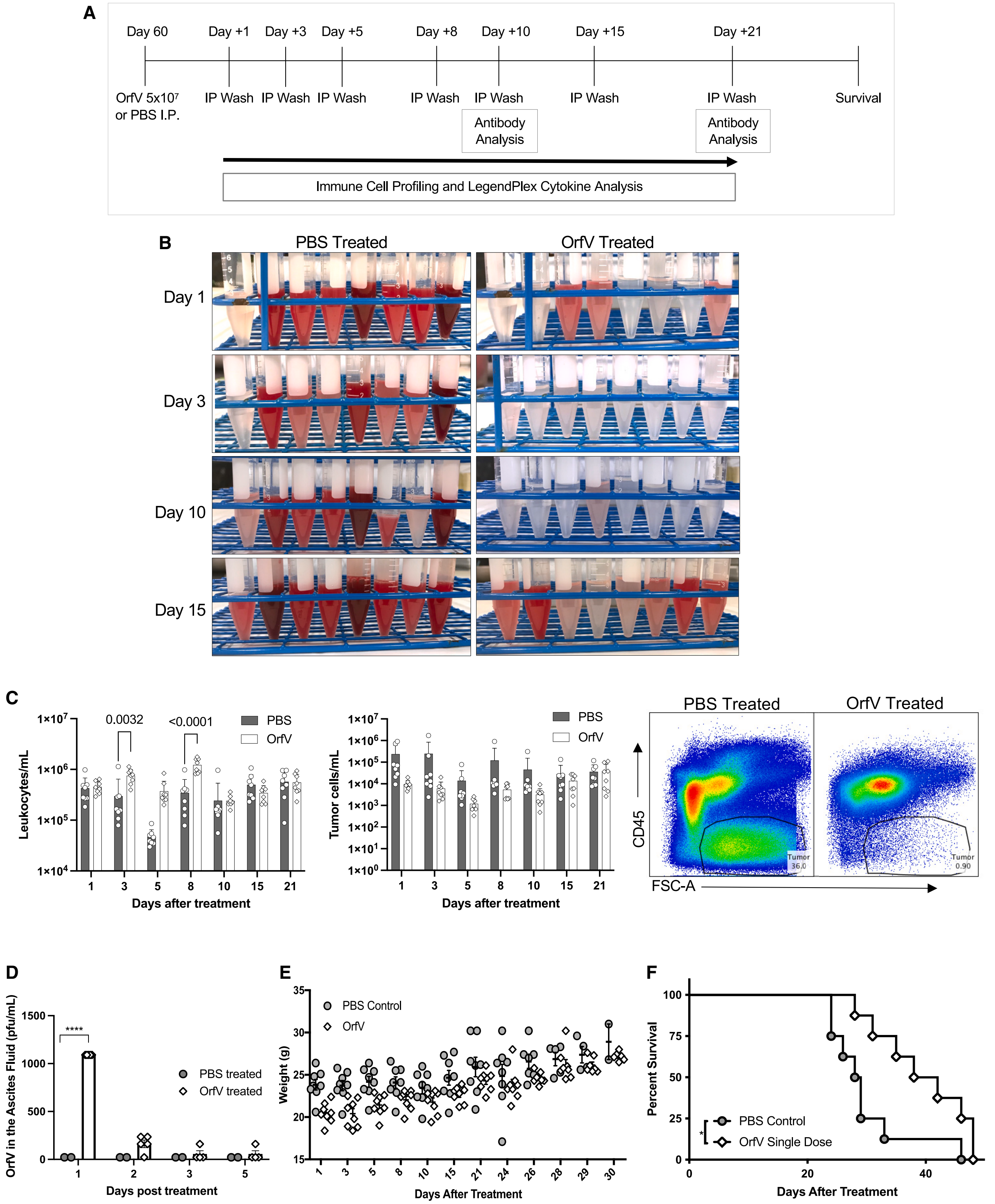

(legend on next page)

(legend on next page)

Here, we endeavored to further dissect the mechanism by which OrfV treatment extends survival in a preclinical model of advanced-stage ovarian cancer that mirrors advanced-stage EOC in humans. We sampled ascites fluid, which contains a variety of cellular and acellular components known to contribute to metastasis and chemoresistance, on days 1, 3, 5, 8, 10, 15, and 21 after a single intraperitoneal administration of 5e7 plaque-forming units (PFUs) OrfV and analyzed innate (NK, DC, macrophage, neutrophil) and adaptive (CD4 and CD8 T cell) immune cell profiles to characterize the immune response in the TME over time. In addition, we characterized cytokine profiles and antitumor antibody responses in the ascites samples. We found that neutrophils rapidly infiltrated the peritoneal cavity in response to OrfV followed by activated NK cells. Classical ($CD103^{-}$) and cDC1s ($CD103^{+}$) expressing co-stimulatory molecules CD80 and CD40, which are essential for priming tumor-specific cytotoxic T lymphocytes (CTLs),[18] were observed shortly after the peak of the NK cell response. $CD8^{+}$ and $CD4^{+}$ T cells were also elevated within the ascites TME of OrfV-treated mice relative to PBS controls, with peak numbers observed between 5 and 8 days posttreatment. Upon identifying the peak and wane of individual effector cell responses within the TME in relation to tumor burden over time, we identified an optimal time to re-administer OrfV to sustain antitumor immune responses within the TME, which resulted in extended survival compared to a single dose.

These results provide insight into the kinetics of the innate and adaptive immune response to OrfV within the ascites TME. A thorough understanding of the complex pattern of interactions between OrfV and the various cells of the immune system and their functions within the TME is crucial to identifying determinants that are fundamental or detrimental to therapeutic success and will aid in the improved design of treatment protocols and rational selection of therapeutic transgenes to express from recombinant OrfV to drive curative outcomes.

RESULTS

A single dose of OrfV reduces ascites tumor burden, recruits leukocytes to the TME, and extends survival in a preclinical model of advanced EOC

The immunotherapeutic potential of OrfV for ovarian cancer has been tested previously using the syngeneic orthotopic ID8 model.[17] Here, we aimed to further dissect the mechanisms by which OrfV functions as an immunotherapy against preclinical advanced EOC. A total of 1×10^{6} ID8 tumor cells were implanted into the left ovarian bursa of female C57BL/6 mice. After 60 days, mice presented with disease mirroring advanced-stage EOC in humans, including a large primary tumor, accumulation of ascites fluid in the peritoneal cavity, and dissemination of secondary tumor lesions throughout the peritoneal cavity. At 60 days posttumor implantation, ID8 tumor-bearing mice were injected with a single dose of 5×10^{7} PFU of OrfV intraperitoneally and subjected to peritoneal lavage sampling for immune response analysis over time (Figure 1A). Within 24 h, there was a visible reduction in bloody ascites in the OrfV-treated mice compared to PBS-treated mice, and this was sustained for up to 10 days posttreatment (Figure 1B). A concomitant reduction in the number of free-floating tumor cells within the peritoneal cavity was observed in OrfV-treated mice for up to 10 days following OrfV administration, which correlated with an increase in the number of leukocytes (Figure 1C). Infectious OrfV was detected in the ascites fluid 1 day after treatment but waned by day 2 postadministration, suggesting that the reduction in free-floating tumor cells and recruitment of leukocytes to the TME observed following OrfV treatment was sustained in the absence of infectious virus (Figure 1D). Considering that body weight is indicative of ascites accumulation and one of the determinants of endpoint in this model, mouse weights were measured over time. A single dose of OrfV appeared to slow the weight gain in treated mice (Figure 1E) and significantly extended survival compared to PBS-treated mice (Figure 1F). Cumulatively, these data suggested that OrfV was effective in the near absence of viral replication at controlling ascites burden and disease spread by targeting tumor cells and recruiting leukocytes within the ascites TME to improve survival outcomes.

Kinetic analysis of the immune cell profile within the ascites TME following OrfV administration

To further characterize the immunological effects of OrfV therapy, we conducted a comprehensive kinetic analysis of the immune response within the ascites TME following intraperitoneal treatment with PBS or one dose of 5×10^{7} PFU OrfV. Peritoneal lavages were performed on days 1, 3, 5, 8, 10, 15, and 21 following treatment and subjected to immune cell phenotyping by flow cytometry. OrfV stimulated the recruitment and persistence of several effector cell subsets in the ascites TME, namely neutrophils (Figure 2A), NK cells (Figure 2C), DCs (Figures 2D and 2E), $CD8^{+}$ (Figure 2F) and $CD4^{+}$ T cells (Figure 2G). Within 48 h of OrfV administration, neutrophil and NK cell numbers were increased within the peritoneal cavity of

Figure 1. OrfV intervention reduces ascites tumor burden, recruits leukocytes to the TME, and extends survival in a preclinical model of advanced EOC
Female C57BL/6 mice were implanted with 1e+06 ID8 cells in the left ovarian bursa. (A) Mice were treated with PBS or a single dose of 5e+07 PFU OrfV intraperitoneally on day 60 and subjected to peritoneal lavage fluid sampling (immunoprecipitation [IP] wash) on days 1, 3, 5, 8, 10, 15, and 21 days posttreatment for immune response analysis and survival assessment as per the schematic. (B) Image of peritoneal lavage fluid samples collected from ID8 tumor-bearing mice 1, 3, 10, and 15 days after treatment with PBS or OrfV. (C) The number of leukocytes (mean plus standard deviation, n = 8) per milliliter of ascites fluid from mice treated with OrfV or PBS over time (left). The number of tumor cells (mean plus standard deviation, n = 8) per milliliter of ascites fluid from mice treated with OrfV or PBS over time (center). Statistical analyses were performed using 2-way ANOVA. Representative dot plot of the reduction in tumor burden within ascites fluid at 24 h posttreatment is shown (defined by $CD45^{-}$ expression and high FSC-A). (D) Infectious OrfV particles within the ascites fluid was quantified over time by $TCID_{50}$. Statistical analysis was conducted by 2-way ANOVA. (E) Mouse weights were graphed over time following treatment. (F) Survival was assessed by the Mantel-Cox log rank test. Significance was determined by $p \leq 0.05$ (*$p \leq 0.05$; ****$p \leq 0.0001$). FSC-A, forward scatter-area. Graphs show means and SE.

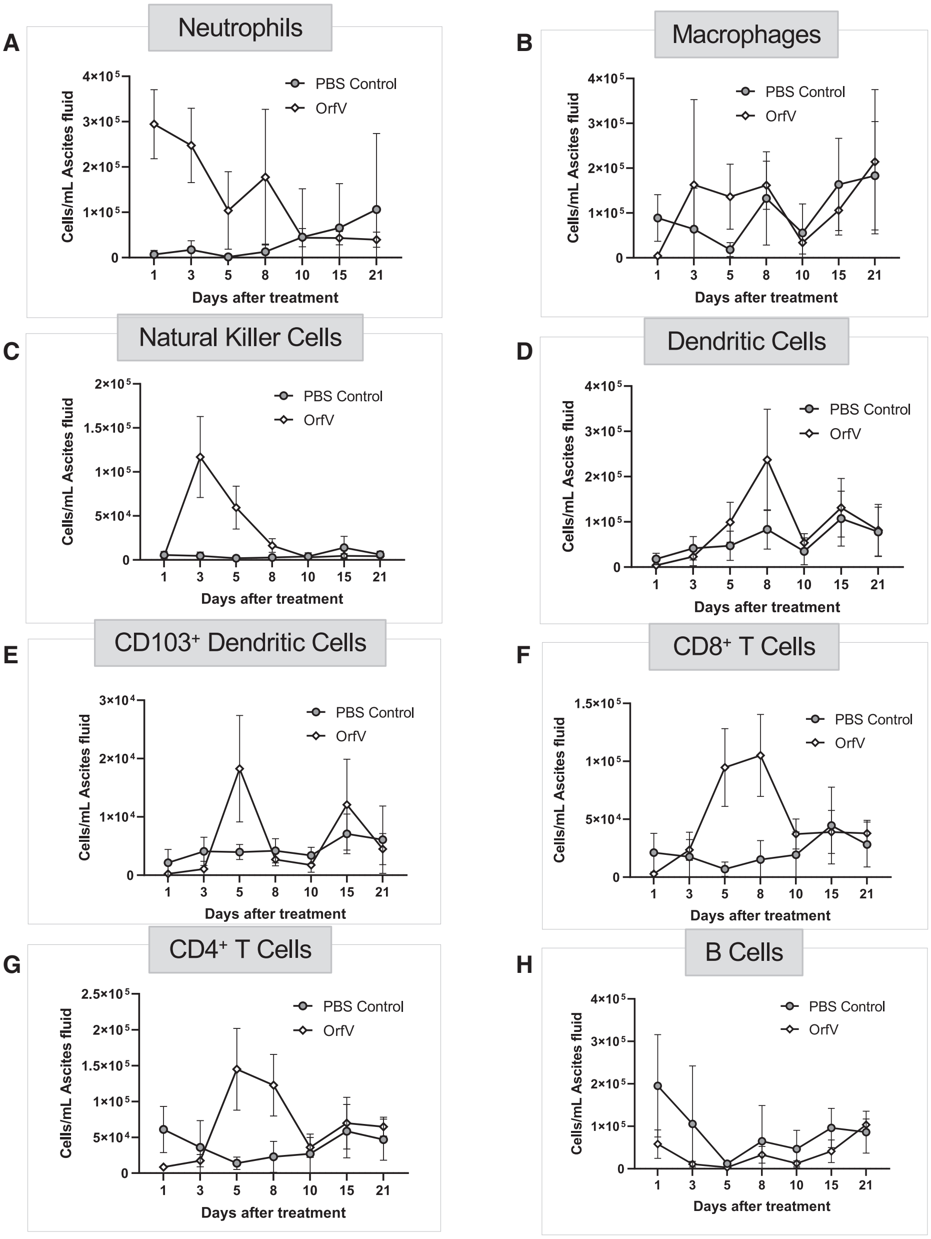

I

Significance values comparing immune cell numbers in the ascites fluid of OrfV and PBS treated tumor bearing mice								
**Days Post Treatment**	**Neutrophils**	**Macrophages**	**Natural Killer Cells**	**Dendritic Cells**	**$CD103^+$ Dendritic Cells**	**$CD8^+$ T Cells**	**$CD4^+$ T Cells**	**B Cells**
**1**	<0.0001	0.427	>0.9999	0.9971	0.9466	0.438	0.0053	<0.0001
**3**	<0.0001	0.244	<0.0001	0.986	0.618	0.9971	0.8299	0.0134
**5**	0.0914	0.0954	<0.0001	0.2353	<0.0001	<0.0001	<0.0001	>0.9999
**8**	0.0007	0.9954	0.3829	<0.0001	0.985	<0.0001	<0.0001	0.9034
**10**	>0.9999	0.9994	>0.9999	0.983	0.9746	0.4669	0.9969	0.8687
**15**	0.9978	0.8321	0.8048	0.9417	0.0869	0.9981	0.9868	0.3887
**21**	0.5424	0.9932	>0.9999	>0.9999	0.978	0.9527	0.8463	0.9968

(legend on next page)

treated mice and remained elevated up to 10 days following treatment (Figures 2A and 2C). The numbers of classical ($CD103^-$) and cDC1s ($CD103^+$), and $CD8^+$ and $CD4^+$ T cells were also elevated within the ascites TME of OrfV-treated mice relative to PBS controls, with peak numbers observed between 5 and 8 days posttreatment (Figures 2D–2G). OrfV treatment did not significantly alter the numbers of macrophages or B cells within the peritoneal cavity (Figures 2B and 2H). A comprehensive heatmap illustrating immune responses over time is shown in Figure 2I. Together, these data indicated that OrfV intervention stimulates an inflammatory cascade wherein multiple effector subsets, including neutrophils, NK cells, DCs, and T cells are recruited to the TME in ID8 tumor-bearing mice.

OrfV therapy enhances activated NK cell and DC effector phenotypes within the ascites TME

OrfV is known to stimulate potent antitumor NK cell responses,[19] which we have previously shown as playing an important role in mediating OrfV therapeutic efficacy in conjunction with cDC1s in ID8 tumor-bearing mice.[20] To further characterize the NK cell and DC response elicited by OrfV, peritoneal lavages were conducted at distinct time points posttreatment. Phenotypic analysis of NK cells in the peritoneal cavity revealed an increase in NK cells expressing the activation markers CD69 (Figure 3A) and programmed death-ligand 1 (PD-L1) (Figure 3C) within 48 h of OrfV therapy, for up to 5 days posttreatment. OrfV did not appear to significantly alter the number or proportion of PD-1^+ NK cells in the peritoneal cavity (Figure 3B). Shortly after the peak of the NK cell response, an increase in the number and proportion of $CD40^+$ $CD80^+$ classical DCs (Figure 3D) and $CD103^+$ cDC1s (Figure 3E) was observed in the peritoneal cavity of OrfV-treated mice relative to PBS controls. These observations demonstrate the ability of OrfV to activate NK and DC effector phenotypes in the ascites TME for a duration of 5 days before there is a return to baseline.

OrfV therapy enhances the recruitment of activated $CD8^+$ T cells to the ascites TME

Following up on our observation of increased $CD8^+$ T cell recruitment to the ascites TME after OrfV therapy, we aimed to further dissect the anticancer CTL response elicited by OrfV. Analysis of $CD8^+$ T cells in the peritoneal cavity revealed an increase in $CD8^+$ T cells expressing the activation markers CD69 (Figure 4A), programmed cell death protein 1 (PD-1) (Figure 4B), and PD-L1 (Figure 4C) within 5 days following OrfV therapy. To determine the contribution of the CTL response to OrfV efficacy against advanced ID8 EOC, peritoneal lavages were performed around the peak of the $CD8^+$ T cell response 10 days following treatment. Using a coculture assay with interferon (IFN)-stimulated ID8 cells to increase major histocompatibility complex (MHC) expression, we observed an increase in the proportion of IFN-γ^+ $CD8^+$ T cells within the peritoneal cavity compared to peripheral blood in both PBS- and OrfV-treated mice (Figure 4D). Interestingly, there were more IFN-γ^+ tumor necrosis factor-α^+ (TNF-α^+) $CD8^+$ T cells in the peritoneal cavity of PBS-treated mice than in the peripheral blood of both PBS- and OrfV-treated mice (Figure 4D), suggesting that OrfV efficacy in the ID8 model may not be $CD8^+$ T cell mediated. Taken together, these data indicated that although OrfV enhances the recruitment of activated $CD8^+$ T cells to the ascites TME, tumor-directed CTL responses do not appear to be enhanced by OrfV therapy. However, further analysis of the tumor-directed CTL response at different time points is required because it is possible that we missed the peak of the T cell response.

OrfV enhances anticancer $CD4^+$ T cell activity in the ascites TME

We similarly assessed the contribution of $CD4^+$ T cell responses to OrfV-mediated anticancer effects and observed an increase in $CD4^+$ T cells expressing the activation markers CD69 (Figure 5A), PD-1 (Figure 5B), and PD-L1 (Figure 5C) within 5 days following OrfV therapy. An increase in ID8 tumor-specific recall responses was also seen from $CD4^+$ T cells isolated from the peritoneal lavage fluid of OrfV-treated mice but not control animals, based on the increased expression of IFN-γ and IFN-γ and TNF-α (Figure 5D). These data indicated that OrfV treatment can stimulate tumor-directed $CD4^+$ T cell responses in the ascites TME.

OrfV intervention is not therapeutically limited by viral-neutralizing antibodies, and induces robust tumor-directed antibody responses

The potential of OVs to elicit antitumor antibody responses has been understudied.[21] To interrogate the contribution of the humoral response to OrfV antitumor efficacy, immunological analyses were extended to include the quantification of tumor-associated antibodies in the plasma and cell-free ascites fluid of OrfV- or PBS-treated mice 10 and 21 days following treatment. Peritoneal lavage fluid and plasma from untreated tumor-free mice were included as a control to assess any potential preexisting cross-reactive antibodies. Diluted plasma and cell-free ascites fluid samples were co-incubated with target ID8 cells and therapy-induced tumor-associated antibodies detected by flow cytometry using a fluorochrome-conjugated anti-mouse immunoglobulin G (IgG) secondarily. An increase in tumor-associated antibodies was detected in both the plasma and cell-free ascites fluid of OrfV-treated mice relative to control animals (Figure 6A). Due to the immunogenic nature of viruses, OVs can

Figure 2. Kinetic analysis of the immune cell profile within the ascites TME following OrfV administration

ID8 mice were treated with 1 dose 5×10^7 PFU OrfV or PBS intraperitoneally on day 60. Ascites fluid was harvested over time for immune cell phenotyping by flow cytometry. The number of innate and adaptive effector cells of the immune response within the peritoneal cavity following treatment was quantified over time by flow cytometry, including (A) neutrophils, (B) macrophages, (C) NK cells, (D) classical DCs, (E) cDC1 ($CD103^+$), (F) $CD8^+$ T cells, (G) $CD4^+$ T cells, and (H) B cells. Graphs depict the mean ± standard deviation of the number of cells per milliliter of ascites fluid for PBS- versus OrfV-treated mice. (I) p values indicating significant differences in the number of a specific cell type between PBS-treated and OrfV-treated mice (*$p \leq 0.05$; **$p \leq 0.01$; ***$p \leq 0.001$; ****$p \leq 0.0001$). Statistical analyses were conducted using 2-way ANOVA. Graphs show means and SE.

Natural Killer Cells

A

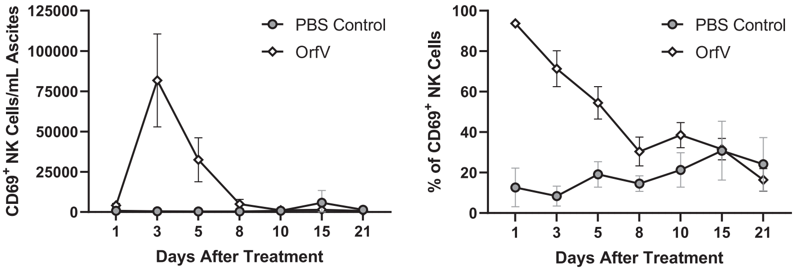

Days Post Treatment	$CD69^+$ NK/mL	% of $CD69^+$ NK
1	0.9825	<0.0001
3	<0.0001	<0.0001
5	<0.0001	<0.0001
8	0.9195	0.0012
10	>0.9999	0.0003
15	0.9408	>0.9999
21	>0.9999	0.3404

Significance between OrfV and PBS treated mice

B

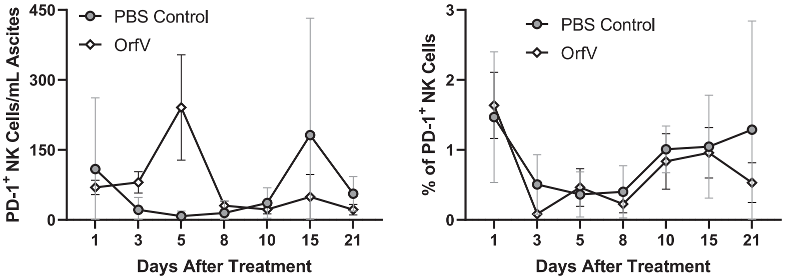

Significance between OrfV and PBS treated mice

Days Post Treatment	$PD\text{-}1^+$ NK/mL	% of $PD\text{-}1^+$ NK
1	0.9591	0.9977
3	0.749	0.7121
5	<0.0001	>0.9999
8	0.9999	0.9968
10	>0.9999	0.9971
15	0.0211	>0.9999
21	0.9819	0.0892

C

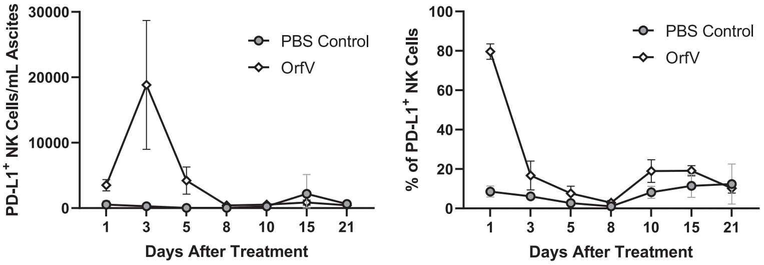

Significance between OrfV and PBS treated mice

Days Post Treatment	$PD\text{-}L1^+$ NK/mL	% of $PD\text{-}L1^+$ NK
1	0.238	<0.0001
3	<0.0001	<0.0001
5	0.0276	0.1933
8	>0.9999	0.9752
10	>0.9999	<0.0001
15	0.952	0.0068
21	>0.9999	0.9414

Dendritic Cells

D

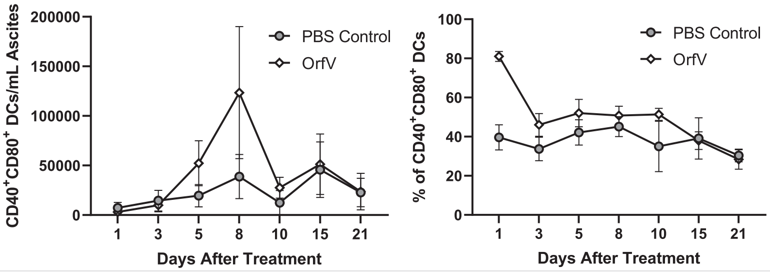

Significance between OrfV and PBS treated mice

Days Post Treatment	$CD40^+$ $CD80^+$ DC/mL	% of $CD40^+$ $CD80^+$ DC
1	0.9999	<0.0001
3	0.9998	0.0019
5	0.0564	0.0219
8	<0.0001	0.4595
10	0.8161	<0.0001
15	0.9994	0.9999
21	>0.9999	0.9958

CD103+ Dendritic Cells

E

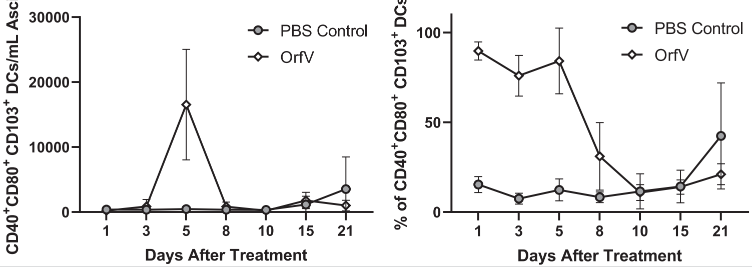

Significance between OrfV and PBS treated mice

Days Post Treatment	$CD40^+$ $CD80^+$ $CD103^+$ DC/mL	% of $CD40^+$ $CD80^+$ $CD103^+$ DC
1	>0.9999	<0.0001
3	0.9998	<0.0001
5	<0.0001	<0.0001
8	0.9999	0.0019
10	>0.9999	>0.9999
15	0.9993	>0.9999
21	0.3696	0.0044

(legend on next page)

stimulate undesired virus-associated immune responses that can result in viral clearance and limit therapeutic efficacy upon re-administration. This can be a particular problem for OV-based platforms, because these often require multiple administrations to achieve optimal therapeutic effects.[22] As such, to investigate the induction of potential therapy-limiting antibody responses, we also subjected diluted plasma and cell-free ascites fluid samples to incubation with OrfV-infected Vero cells for the detection of OrfV-directed antibodies. OrfV-directed antibodies were identified in the plasma at 10 and 21 days following OrfV therapy but appeared delayed in formation within the peritoneal cavity (Figure 6B). To discern the viral neutralization capacity of the OrfV-directed antibodies identified, OrfV was incubated with serial dilutions of plasma and cell-free ascites fluid samples from PBS-treated, OrfV-treated, and untreated tumor-free mice for 1 h before infection of ID8 target cells. Despite co-incubation of OrfV with viral-associated antibodies, OrfV was still able to induce the cell death of target ID8 cells, as evidenced by the reduction in ID8 cell metabolic activity 48 h after incubation (Figure 6C), indicating a lack of virus neutralizing antibodies in the serum and ascites samples. Taken together, these results suggest that OrfV intervention stimulates potent tumor-associated antibody responses and is not therapeutically limited by virus-neutralizing antibodies.

OrfV therapy induces a complex cytokine response within the ascites TME, indicative of a multieffector immune response

Proinflammatory cytokines are known to elicit potent antitumor activities[23]; as such, we sought to characterize the impact of OrfV therapy on the cytokine profile within the ascites TME over time. Peritoneal lavage fluid samples harvested from ID8 tumor-bearing mice at multiple time points after intraperitoneal administration of OrfV or PBS were evaluated using a flow cytometry–based LegendPlex (Biolegend) assay for cytokine and chemokine quantification. Within 24 h following OrfV administration, several proinflammatory cytokines involved in stimulating leukocyte proliferation, recruitment, activation, and effector function were upregulated within the ascites TME compared to PBS controls, the concentrations of which remained sustained up to 8 days post-OrfV (Figure 7A). In particular, significant increases in the concentrations of IFN-γ, monocyte chemoattractant protein-1 (MCP-1), TNF-α, regulated upon activation, normal T cell expressed and presumably secreted (RANTES) chemokine, interferon-γ-inducible protein 10 kDa (IP-10), interleukin-6 (IL-6), and IL-12p70 were observed (Figures 7B and S2). These data indicate that cells within the ascites TME rapidly respond to OrfV by inducing a complex cytokine gradient that may contribute toward the antitumor efficacy of OrfV.

Monitoring leukocyte-to-tumor ratios over time can be used to predict optimal re-administration timing to enhance therapeutic efficacy

Given the observation that several immune effector cells responded to OrfV therapy, we set out to profile the response of individual populations in relation to tumor cell burden in the peritoneal cavity of ID8 tumor-bearing mice following treatment over time. Within 24 h of OrfV administration, an increase in the total number of leukocytes relative to tumor cells was observed within the ascites TME of treated mice, which remained as such up to 15 days following treatment (Figure 8A), correlating with the sustained reduction in tumor cell burden seen post-OrfV (Figure 8B). Further kinetic analysis of individual immune cell populations revealed an increase in the numbers of each effector leukocyte relative to tumor cells within the ascites fluid of OrfV-treated mice (Figures 8C–8J), emphasizing the ability of OrfV to stimulate immune responses that serve to reduce tumor burden. Intriguingly, most immune responses modulated by OrfV waned 15 days post-treatment. At this time, control and OrfV-treated mice had similar leukocyte-to-tumor cell ratios, suggesting a loss of tumor immune control by day 15 posttreatment. Given that repeat dosing has proven crucial for the success of many OVs and evidence of a lack of OrfV-neutralizing antibodies, we hypothesized that administering a second dose of OrfV at the resolution of specific leukocyte responses, or at the time of relapse when all leukocyte-to-tumor ratios have normalized, may serve to extend survival further based on extending OrfV-induced antitumor immune responses. Compared to controls, or mice that received a single dose of OrfV on day 60, survival was significantly enhanced in mice that received a second dose of OrfV intraperitoneally on day 65, correlating with the resolution of the NK response peak and where the highest numbers of leukocytes relative to tumor cells within the peritoneal cavity were observed (Figure 8K). Similarly, survival was extended relative to control or in mice that received a single dose of OrfV on day 60, and in mice that received a second dose of OrfV on day 70 at the resolution of the T cell response peak (Figure 8L) or on day 75 at the time of relapse (Figure 8M). Interestingly, redosing on day 70, when most leukocyte-to-tumor ratios begin to wane, appeared the most beneficial to OrfV therapeutic efficacy because this was the only re-administration or booster group that had significantly increased survival compared to a single dose of OrfV. Taken together, these data suggest that monitoring leukocyte kinetics in relation to tumor burden could provide insight into the optimal time to redose an OV to sustain antitumor immune responses and therapeutic efficacy following OV treatment and suggest that waiting for relapse is too late.

Figure 3. OrfV leads to an increase in activated NK cell and DC effector phenotypes within the ascites TME

Ascites fluid was harvested from ID8 tumor-bearing mice treated with PBS or 5×10^7 PFU OrfV by peritoneal lavage at defined time points posttreatment and subjected to immune cell phenotyping by flow cytometry. The number and proportion of NK cells expressing (A) CD69, (B) PD-1, and (C) PD-L1 in the peritoneal cavity of ID8 mice after treatment was quantified. The number and proportion of CD40$^+$ CD80$^+$ classical DCs (D) and cDC1 (CD103$^+$s) (E) in the peritoneal cavity of ID8 mice were quantified over time following treatment. Graphs depict the mean ± standard deviation of the number of cells per milliliter of ascites fluid for PBS- versus OrfV-treated mice. Tables containing respective p values indicate significant differences in the number of a specific cell type between PBS-treated and OrfV-treated mice (*p $\leq$ 0.05; **p $\leq$ 0.01; ***p $\leq$ 0.001; ****p $\leq$ 0.0001). Statistical analyses were conducted using 2-way ANOVA. Graphs show means and SE.

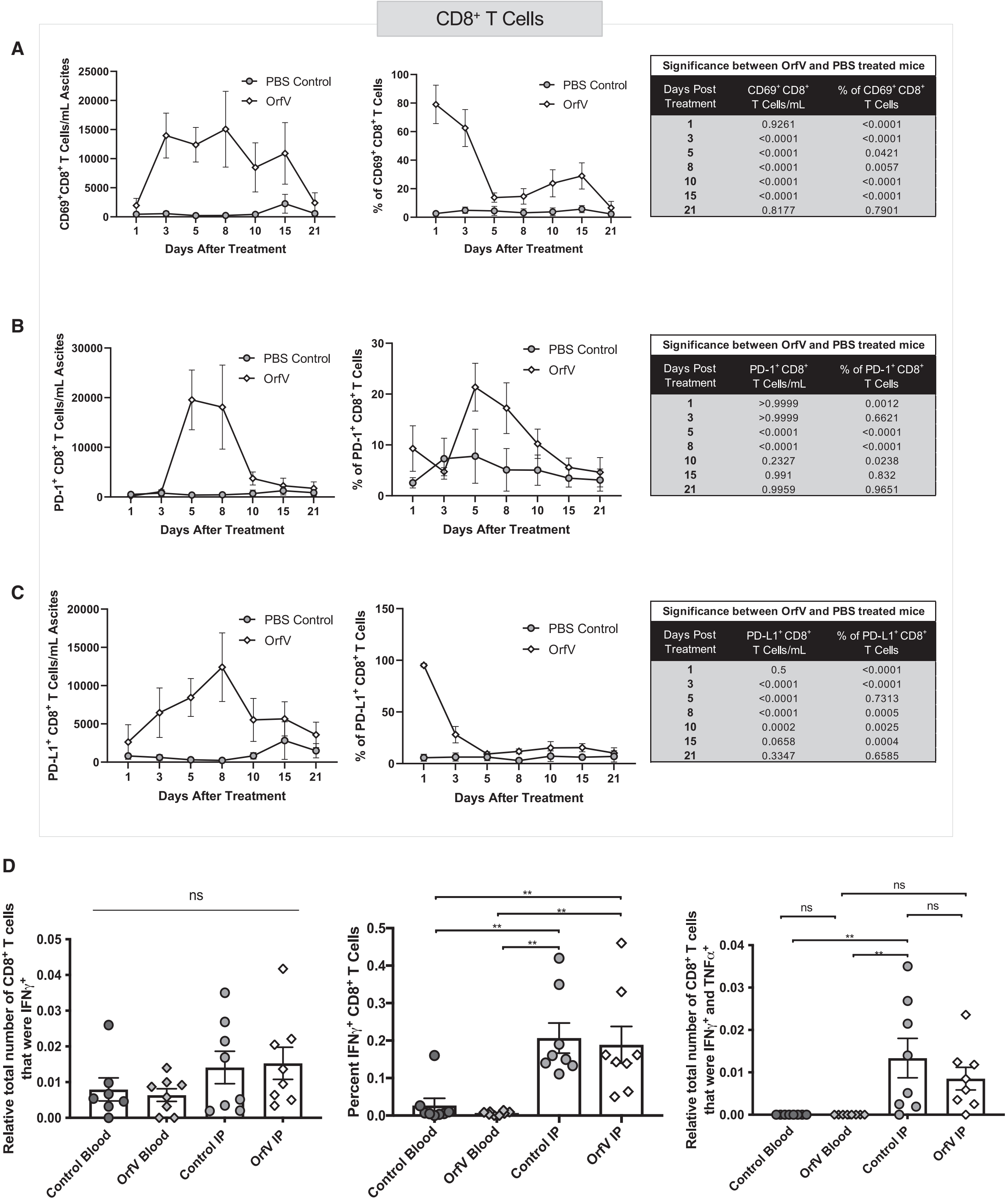

Significance between OrfV and PBS treated mice

Days Post Treatment	$CD69^+$ $CD8^+$ T Cells/mL	% of $CD69^+$ $CD8^+$ T Cells
1	0.9261	<0.0001
3	<0.0001	<0.0001
5	<0.0001	0.0421
8	<0.0001	0.0057
10	<0.0001	<0.0001
15	<0.0001	<0.0001
21	0.8177	0.7901

Significance between OrfV and PBS treated mice

Days Post Treatment	$PD\text{-}1^+$ $CD8^+$ T Cells/mL	% of $PD\text{-}1^+$ $CD8^+$ T Cells
1	>0.9999	0.0012
3	>0.9999	0.6621
5	<0.0001	<0.0001
8	<0.0001	<0.0001
10	0.2327	0.0238
15	0.991	0.832
21	0.9959	0.9651

Significance between OrfV and PBS treated mice

Days Post Treatment	$PD\text{-}L1^+$ $CD8^+$ T Cells/mL	% of $PD\text{-}L1^+$ $CD8^+$ T Cells
1	0.5	<0.0001
3	<0.0001	<0.0001
5	<0.0001	0.7313
8	<0.0001	0.0005
10	0.0002	0.0025
15	0.0658	0.0004
21	0.3347	0.6585

(legend on next page)

DISCUSSION

Oncolytic immunotherapy relies on the ability of an OV to selectively infect tumor cells leading to the release of cellular tumor-associated antigens, danger-associated molecular patterns, and viral pathogen-associated molecular patterns, resulting in the induction of an inflammatory immune response and the recruitment of immune cells to the tumor.[5,24] To our knowledge, this study is the first to map the immune response to OrfV over time in the context of preclinical advanced-stage ovarian cancer. Our results show that a single dose of intraperitoneally administered OrfV was effective at recruiting multiple immune cell populations to the ascites TME, reducing ascites tumor burden, and extending survival relative to controls despite exhibiting a limited production of new virus particles within the peritoneal cavity. Neutrophils were one of the first leukocytes observed to rapidly infiltrate the peritoneal cavity in response to OrfV. Following peak neutrophil activity, increased numbers of NK cells expressing markers associated with activation and effector function were observed in OrfV-treated mice, suggesting that neutrophils may be involved in the recruitment of NK cells to the TME, as has been previously documented.[19,20,25] Both classical (CD103$^-$) and cDC1s (CD103$^+$) with increased expression of the co-stimulatory molecules CD80 and CD40, essential for priming tumor-specific CTLs,[18] were observed in increased numbers in OrfV-treated mice shortly thereafter the peak of the NK response. These results support our previously established finding that NK cells support DC recruitment and activity following OrfV administration[17,21] and align with previous reports that highlight the importance of DC-NK cross-talk mechanisms in enhancing antitumor immunity following OV treatment.[22]

Within the ascites TME of OrfV-treated mice, IFN-γ and TNF-α, which are involved in NK and T cell tumor cell cytotoxicity,[26] MCP-1, IP-10 and RANTES, which are involved in leukocyte proliferation and recruitment,[21,27] and IL-6 and IL-12p70, which are involved in anti-tumor Th-1 stimulation,[28] were significantly increased relative to controls. As a testament to their importance in strengthening the antitumor immune response, a variety of OVs have been engineered to express these exact proinflammatory cytokines, resulting in enhanced antitumor immunity.[29–32] Future directions include investigating the significance of each cytokine, potentially via immune depletion studies, to determine key contributors to OrfV-mediated antitumor immunity. These critical immune pathways can then be potentiated by engineering OrfV to express key cytokines that will further enhance efficacy by shaping a more appropriate immune stimulatory environment in the TME.

It should be noted that various subsets of immunological effector cells, such as neutrophils, macrophages, and B cells, can exhibit function in either cancer suppression or progression,[26,33,34] making the functional relevance of their persistence within the TME following OrfV difficult to discern. Throughout the course of our study, the proportion and persistence of macrophages and B cells within the ascites TME remained relatively unchanged between OrfV- and PBS-treated mice, which could be evidence of preexisting tumor-promoting cell populations. On the one hand, classically activated macrophages (M1) play important roles in host defense and tumor cell killing through their potent phagocytic capabilities and their ability to produce proinflammatory cytokines and reactive oxygen/nitrogen species.[26,34] On the other hand, M2-polarized macrophages not only produce anti-inflammatory cytokines and suppress the immunosurveillance against tumor cells but they also promote angiogenesis and matrix remodeling to promote tumor progression and metastasis.[26,34] The bulk depletion of macrophages in murine peritoneal ovarian cancer models has previously been shown to suppress cancer progression and reduce the accumulation of ascites, suggesting that M2 macrophages may play a more significant role in preclinical ovarian cancer progression.[35,36] Similarly, B cells can produce cytokines or act as antigen-presenting cells to improve tumor-specific CTL activity and directly improve antitumor immunity through the production of tumor-associated antibodies or exhibit protumorigenic activity through the secretion of anti-inflammatory cytokines.[26] It will therefore be essential for future studies to conduct more detailed immunophenotyping of immune cell subsets and their affiliated cytokine secretion profiles within the TME to discern pro- versus antitumor activity within the ascites TME and following OrfV therapy. A limitation of this study was the fact that we were unable to include groups comprising non-tumor-bearing mice treated with either PBS or OrfV in our analysis due to the logistical difficulties associated with running concurrent and frequent flow cytometry experiments with such large numbers of samples. Therefore, because there was not a non-tumor control built into the experimental method, we cannot be certain that the model truly reflects TME-associated changes. It is possible that OrfV may be producing a nonspecific peritonitis that limits the tumor growth in this model.

Figure 4. OrfV treatment leads to an increase in activated CD8$^+$ T cells in the ascites TME of ID8 tumor-bearing mice

ID8 tumor-bearing mice were treated with 5×10^7 PFU OrfV or PBS on day 60 posttumor cell implantation. Ascites fluid was harvested by peritoneal lavage at defined time points and subjected to immune cell phenotyping by flow cytometry. The number and proportion of CD8$^+$ T cells expressing (A) CD69, (B) PD-1, and (C) PD-L1 in the peritoneal cavity following treatment were quantified. Graphs depict the mean ± standard deviation of the number of cells per milliliter of ascites fluid for PBS- versus OrfV-treated mice. Tables containing respective p values indicate significant differences in the number of a specific cell type between PBS-treated and OrfV-treated mice (*$p \leq 0.05$; **$p \leq 0.01$; ***$p \leq 0.001$; ****$p \leq 0.0001$). Statistical analyses were conducted using 2-way ANOVA. Ten days after treatment, blood was collected by nonlethal retroorbital bleed, and peritoneal lavage fluid was harvested for the flow cytometric analysis of tumor-specific T cell responses using a coculture assay with IFN-stimulated target ID8 cells. (D) The number and proportion of tumor-specific IFN-γ^+ CD8$^+$ T cells (left, center) and IFN-γ^+ TNFα^+ CD8$^+$ T cells (right) in the peritoneal cavity following treatment were quantified by flow cytometry. Statistical analyses were performed by 2-way ANOVA. Significance was determined by $p \leq 0.05$ (**$p \leq 0.01$; ****$p \leq 0.0001$). ns, not significant. Graphs show mean and SE.

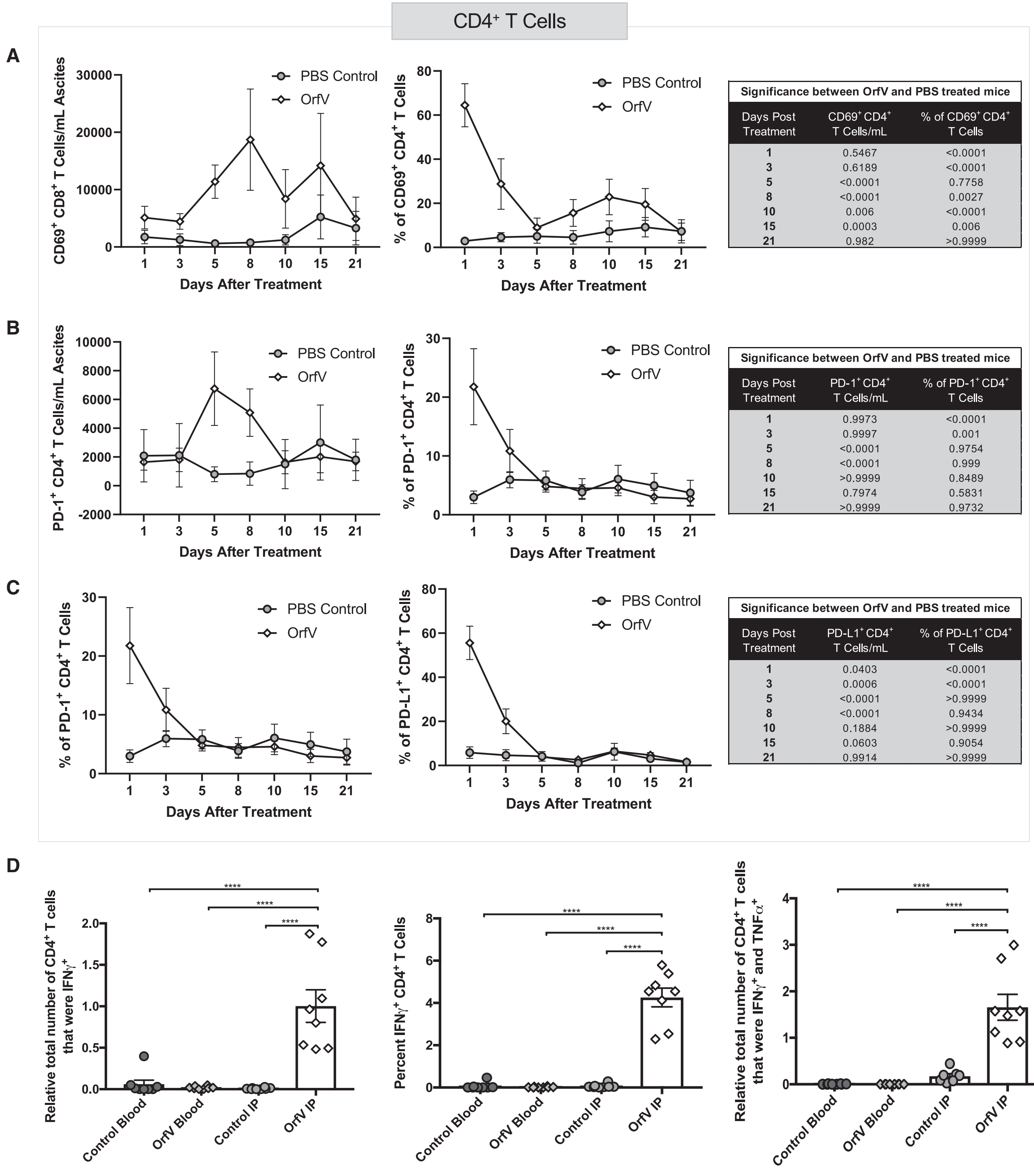

Figure 5. OrfV treatment stimulates tumor-associated CD4$^+$ T cell responses in the ascites TME

ID8-tumor bearing mice were subjected to intraperitoneal administration of 5×10^7 PFU OrfV or PBS on day 60 posttumor cell implantation, and ascites fluid was harvested by peritoneal lavage at defined time points and subjected to immune cell phenotyping by flow cytometry. The number and proportion of CD4$^+$ T cells expressing (A) CD69, (B) PD-1, and (C) PD-L1 in the peritoneal cavity following were quantified. Graphs depict the mean ± standard deviation of the number of cells per milliliter of ascites fluid for PBS- versus OrfV-treated mice. Tables containing respective p values indicate significant differences in the number of a specific cell type between PBS-treated and OrfV-treated

(legend continued on next page)

CD8$^+$ CTLs have long been regarded as the major subset of T cells responsible for mediating cancer cell cytotoxicity and long-term antitumor immunity following immunotherapy treatment. OrfV intervention led to an increase in the proportion of CD4$^+$ and CD8$^+$ T cells within the ascites TME with enhanced expression of inflammatory cytokines and markers involved in activation and antitumor function. Despite this, OrfV therapy did not appear to enhance tumor-specific CTL responses. However, OrfV intervention did augment tumor-directed CD4$^+$ T cell responses within the ascites TME. The insufficient generation of tumor-specific CTLs following OrfV therapy may be as a result of a number of factors that we did not investigate in this study, including insufficient neoantigen load in the ID8 EOC model, insufficient MHC class I expression by ID8 cells, weak CTL priming to subdominant ID8 epitopes due to strong priming against immunodominant OrfV protein epitopes, or the fact that we may have missed the peak of the T cell response. A derivative of the murine ovarian ID8 model has previously been targeted with neoantigen vaccines that increased the proportion of activated CD4$^+$ and CD8$^+$ T cells but did not result in T cell–mediated tumor control due to insufficient MHC-presented neoantigen epitopes.[37] Modifying OrfV to express ID8 tumor antigens may be a logical next step to maximize the number of activated tumor-specific CTLs within the ascites TME. There is limited cause for concern over CTL priming to tumor epitopes being suppressed as a result of antiviral CTL priming, given that researchers have showcased a short-lived duration of OrfV-specific cellular immunity.[12,38] Recently, stimulated splenocytes from mice immunized with ovalbumin-expressing OrfV exhibited strong SIINFEKL-specific CTL responses, with limited responses against OrfV-derived MHC peptides.[39] Nevertheless, it will be important to determine the magnitude of OrfV-specific CD8$^+$ T cell responses and their impact on repeat dosing and therapeutic outcomes.

The upregulation of PD-1 and/or PD-L1 on both NK cells and CD4$^+$ and CD8$^+$ T cells following OrfV administration suggests that combining OrfV with immune checkpoint blockade may enhance anticancer NK and T cell responses, as has been observed with other OVs.[40] Unfortunately, we have previously observed a lack of synergy with anti-PD-1 in this model (despite ID8 cells expressing PD-L1[17]), indicating that PD-1-driven suppression of T cells and/or NK cells may be negligible in this model or that it does not negatively affect OrfV-mediated therapy.[17] Further studies are needed to characterize the relevance of immune checkpoint blockade in ovarian cancer in the context of NK and T cell responses mediated by OrfV intervention.

The role of tumor-associated antibodies in the efficacy of OV therapy has been understudied, likely due to overshadowing by the effort to generate tumor-specific T cell responses and the lack of effective tools for assessment. Using a flow cytometry-based assay, antibodies against bulk ID8 tumor antigens were detected in increased amounts in the circulation and peritoneal cavity of OrfV-treated mice relative to control animals, suggesting that tumor antibody binding may play a significant role in clearing tumor burden and extending survival following OrfV therapy. This is particularly advantageous, given the capacity of these antibodies to bind multiple tumor targets, reducing the risk of tumor escape by antigen loss. However, the potential for autoreactive antibody production following OrfV therapy requires assessment. In addition, future studies are needed to enhance our understanding of the function of these OrfV-induced tumor-associated antibodies, such as in mediating antibody-dependent cellular cytotoxicity with NK cells, which have been shown to constitute a dominant effector cell type responsible for driving OrfV antitumor efficacy, or by enhancing the phagocytosis, opsonization, or complement-mediated cytotoxicity of tumor cells.

A major limitation of the extensive use of OVs in cancer treatment is the rapid neutralization by the immune system, which can limit viral spread and reduce the efficacy of repeat administrations.[21] In this study, OrfV-associated antibodies were detected in circulation at 10 and 21 days postadministration but were unexpectedly delayed in formation in the peritoneal cavity. The therapeutic effects of OrfV were not limited by these antibodies, a phenomenon that has been reported frequently in the context of OrfV infection.[12,41] The absence of OrfV neutralizing antibodies provides the added advantage of re-administration when peak effector cell responses begin to wane so as to sustain antitumor responses beyond the primary response, which showed results in improved therapeutic outcomes. The mechanisms behind the delay in OrfV-specific antibody formation within the ascites TME are unclear, and it remains to be determined how the magnitude and neutralizing capacity of these antibodies change upon subsequent OrfV administrations.

Our kinetic analysis of immune cell infiltration into the TME demonstrates that most immune cell populations return to baseline by day 15 post-OrfV. From our survival data, only redosing at day 70 was better than a single dose at day 60; redosing at day 75 was not better than a single dose at day 60, leading us to conclude that only redosing at day 70 improved survival. One may have hypothesized that a second dose given at day 15 post-OrfV, when immune cell populations have returned to baseline, would be most efficacious. However, this was not the case. Perhaps boosting with OrfV when there are still somewhat elevated levels of immune cells within the TME leads to more rapid and/or robust recruitment of immune cells, thereby prolonging the antitumor effect. At days post-OrfV, most immune cells have left the TME, but not all. It is possible that the remaining immune cells support a rapid re-activation of immune cell populations. This leads

mice (**$p \leq 0.01$). Statistical analyses were conducted using 2-way ANOVA. Ten days after treatment, blood was collected by nonlethal retroorbital bleed, and peritoneal lavage fluid was harvested for the flow cytometric analysis of tumor-specific T cell responses using a coculture assay with IFN-stimulated target ID8 cells. (D) The number and proportion of tumor-directed IFN-γ^+ CD4$^+$ T cells (left, center) and IFN-γ^+ TNF-α^+ CD4$^+$ T cells (right) in the peritoneal cavity following treatment were quantified by flow cytometry. Statistical analyses were performed by 2-way ANOVA. Significance was determined by $p \leq 0.05$ (****$p \leq 0.0001$). Graphs show mean and SE.

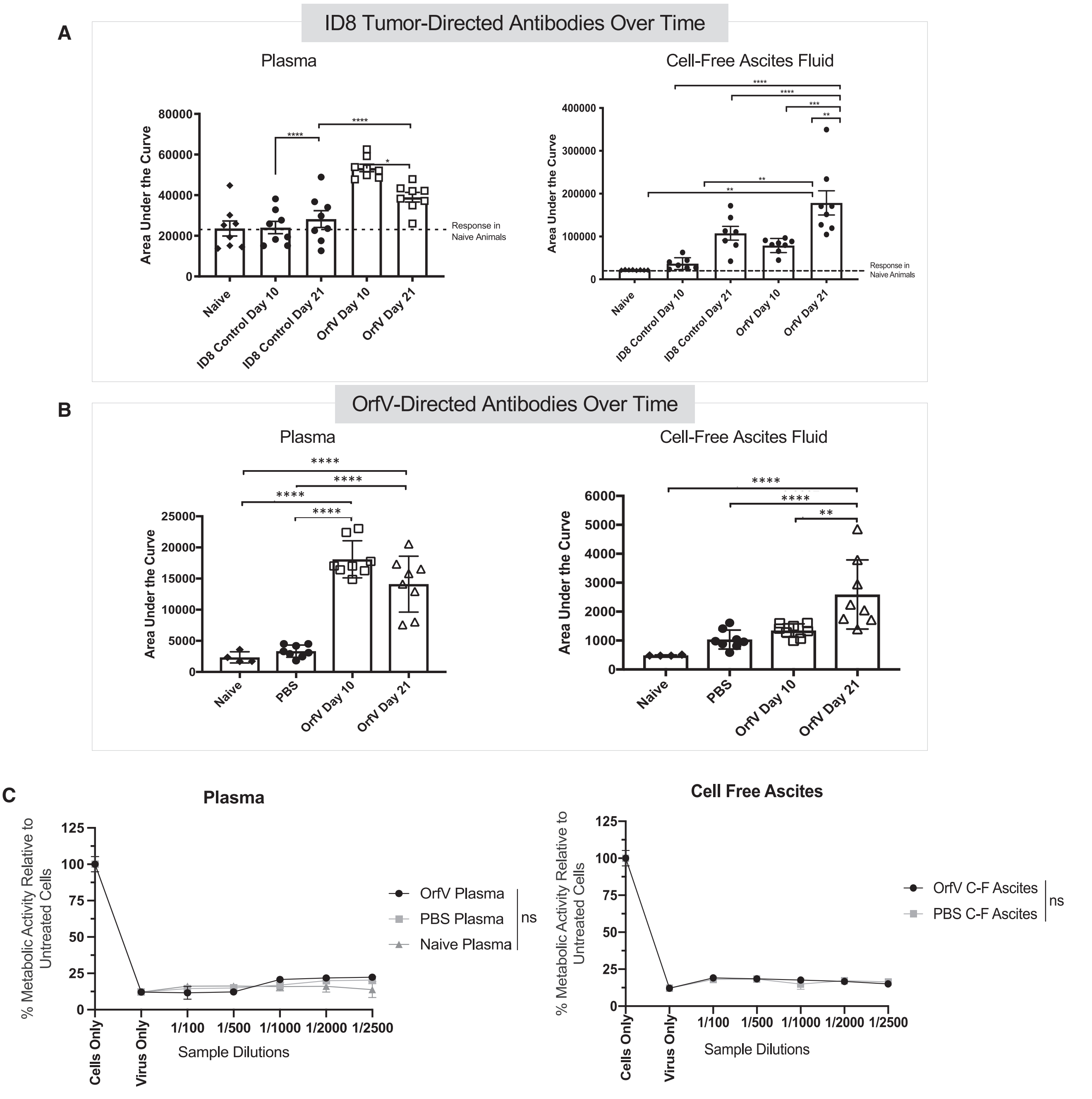

Figure 6. Intraperitoneal administration of OrfV augments tumor-directed antibody responses in the ascites TME

Tumor-associated and OrfV-directed antibody responses were quantified by flow cytometry on HI plasma and cell-free ascites fluid collected from untreated tumor-free naive or ID8 tumor-bearing mice 10 and 21 days following treatment with PBS or OrfV. Tumor-associated and OrfV-directed antibody binding was represented as a function of positive anti-mouse IgG1-Alexa Fluor 488 conjugated secondary antibody signals from target ID8 or OrfV-infected Vero cells. Curves were generated from the mean fluorescence intensity of positive secondary antibody signal from target cells treated with serial dilutions of samples and used to calculate the areas under the curves. The area under the curve was used to assess the (A) tumor-associated antibody response and the (B) OrfV-directed antibody response within the plasma (left) and cell-free ascites fluid (right) of PBS- or OrfV-treated mice. Statistical analysis was conducted using 2-way ANOVA. The virus-neutralizing effect of OrfV-directed antibodies within plasma and cell-free ascites fluid of untreated naive or ID8 mice treated with OrfV or PBS was assessed by viral neutralizing assay. OrfV at an MOI of 3 was incubated with serial dilutions of OrfV-directed antibody-containing plasma and cell-free ascites fluid samples for 1 h before co-incubation with ID8 target cells. (C) ID8 cell viability 48 h after co-incubation was determined by resazurin metabolic activity assay. Data are represented as the percentage of metabolic activity of ID8 cells compared to untreated cell controls. Statistical analysis was by 2-way ANOVA. Significance was determined by $p \leq 0.05$ (*$p \leq 0.05$; **$p \leq 0.01$; ***$p \leq 0.001$; ****$p \leq 0.0001$). Graphs show mean and SE.

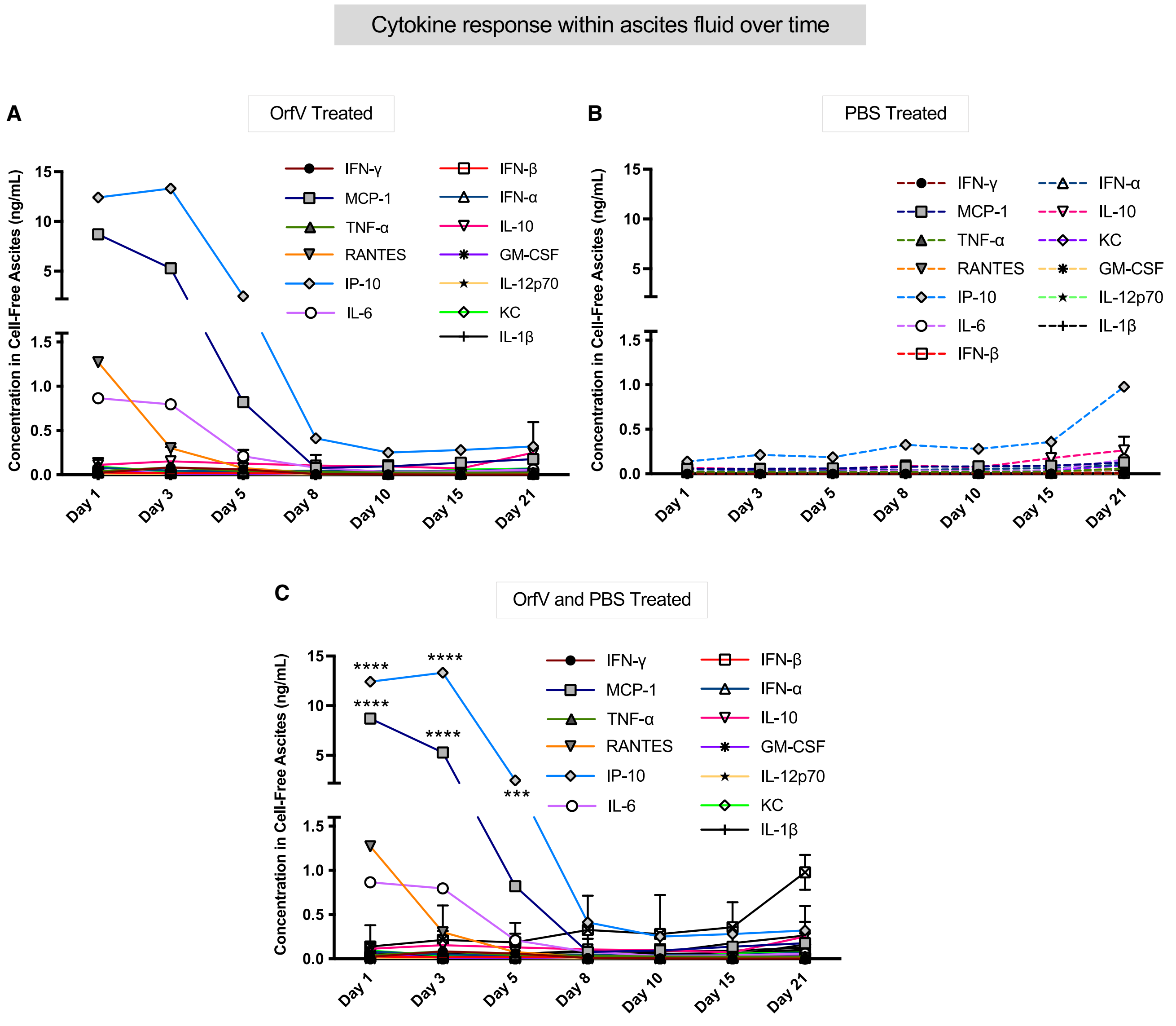

Figure 7. OrfV treatment alters the cytokine profile in the ascites TME of ID8 tumor-bearing mice

Following OrfV or PBS treatment, ascites fluid from ID8 tumor-bearing mice was harvested at defined time points and cytokine concentrations quantified using a LEGENDplex flow cytometry assay. The cytokine concentrations in the peritoneal cavity over time following treatment with OrfV (A) or PBS (B) are shown. (C) Cytokine concentrations for both OrfV- and PBS-treated mice are shown together on the same graph, and statistically significant differences in cytokine concentrations between the OrfV-treated and PBS-treated mice were determined. Data are presented as mean ± SEM, and statistical differences between groups were measured by 2-way ANOVA. Statistical significance was designated as ***$p \leq 0.001$; ****$p \leq 0.0001$. Graphs show mean and SE.

to the observed prolonged survival relative to when booster doses were administered on day 5 post-OrfV, when there is an abundance of immune cells to control OrfV infection, or day 15 post-OrfV, when there may be inadequate numbers of immune cells to stimulate a strong antitumor immune response. Future studies involving detailed immunophenotyping of immune cells that traffic into and out of the TME over time, as well as immune cell depletion studies, are being conducted to determine the exact cell types that contribute to efficacy in this model.

This study is not without its limitations. Although repeat sampling of the ascites fluid has some drawbacks, we reasoned that because we were injecting a higher volume (3 mL) than we were removing (? 1.5 mL), this would not deplete the peritoneal cavity of cells, so cells from day 3 should still be present to affect day 5 and so on. In addition, because we were not preferentially sampling any specific cell type with this method, we would not anticipate that this would change the dynamics of the immune response. Although we admit that there are some disadvantages to the repeat sampling approach, we believe

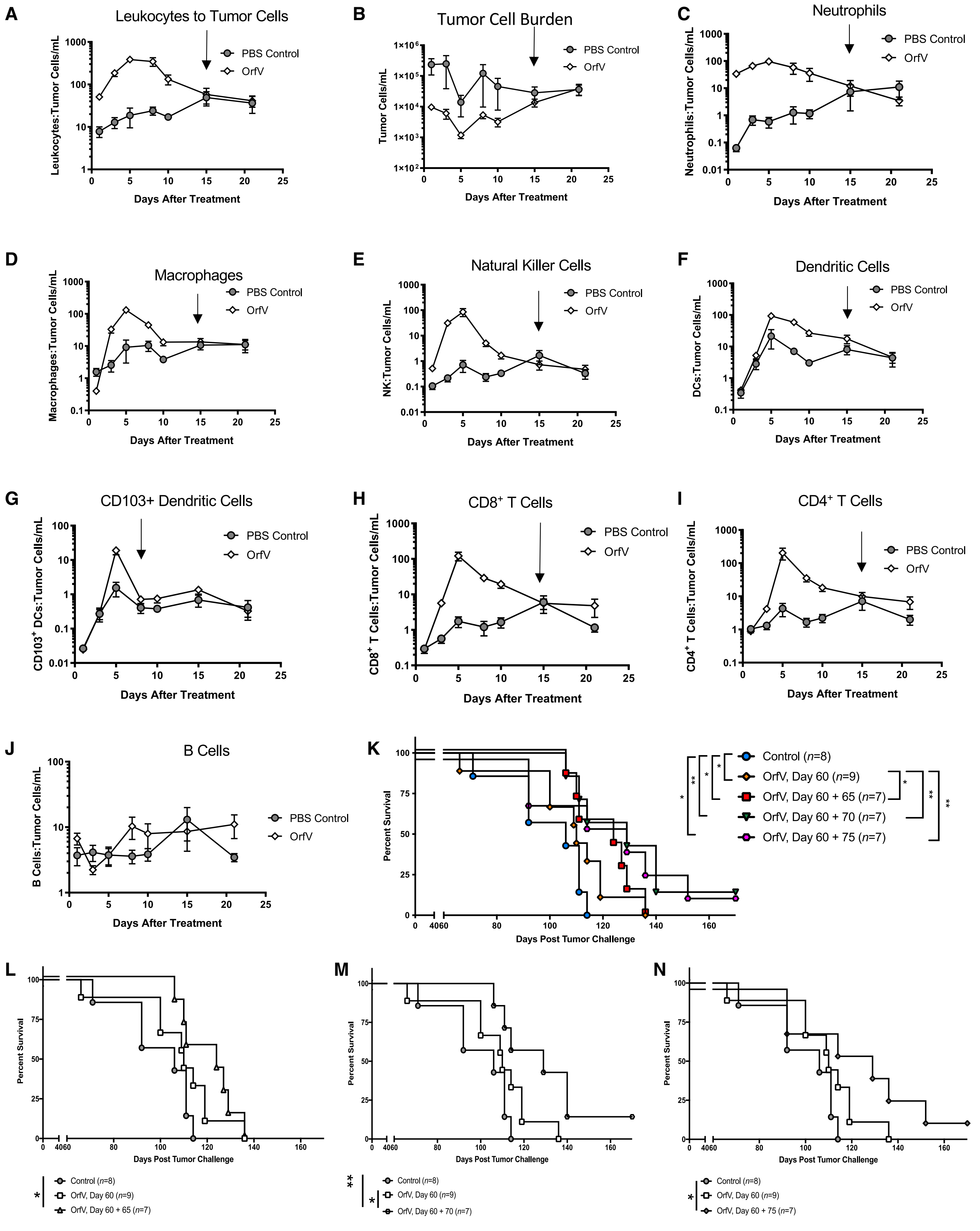

(legend on next page)

that this is outweighed by the benefits of being about to investigate the dynamics of the immune response to tumor and virus over time in the TME in the same animal.

Despite the extensive amount of research remaining to properly understand the mechanisms of OrfV therapy in the context of ovarian cancer, the kinetic analyses performed herein revealed dominant drivers behind OrfV-induced immunity and the time line of responses within the ascites TME, which we showed can be exploited to dictate optimal treatment re-administration times to improve therapeutic outcomes. The results from this study are effective in outlining how kinetic analyses of the immune responses elicited by a given immunotherapy can be used inform the design of future treatment protocols to improve the efficacy of oncolytic viral therapies. These results also provide us with a model for further dissecting the immune response to OrfV and other OVs, because repeated sampling of the TME from the same mice over time is not possible with solid tumors. Finally, the complexity of these data provides several avenues for further exploration of the numerous antitumor effector phenotypes, functions, and mechanisms of action relevant to enhancing OrfV therapeutic outcomes.

MATERIALS AND METHODS

Ethics and biohazard certification

Animal experimentation was approved under animal utilization protocol 3807 by the Animal Care Committee at the University of Guelph, Guelph, Ontario, Canada. All of the experiments were conducted following the guidelines from the Canadian Council on Animal Care. Virus-related research was conducted in certified containment level-2 facilities under biohazard permit A-314-02-24-10 issued by the University of Guelph's Biosafety Committee.

Mice

Eight-week-old female C57BL/6 mice (strain code 027; Charles River Laboratories) were housed four per cage in the animal isolation unit at the University of Guelph under specific pathogen-free conditions. Food and water were provided *ad libitum*. Mice were acclimatized to the facility for 1 week before experimentation.

Cell lines

ID8 transformed murine ovarian surface epithelial cells were generously donated by Drs. K. Roby and P. Terranova (Kansas State University) and were cultured in DMEM (HyClone, catalog no. SH3002201) containing 10% heat-inactivated (HI) fetal bovine serum (FBS). Sheep skin fibroblasts (SSF cells; continuous SSF cell line obtained from Dr. Dusty Miller of the Fred Hutchinson Cancer Research Center)[14] and Vero cells (ATCC-CCL-81) were cultured in DMEM containing 10% FBS. All of the cell lines were cultured in a humidified incubator at 5% CO_2 and 37.0°C and were confirmed to be mycoplasma free before use (MycoAlert PLUS detection kit, Lonza, catalog no. LT07-705).

Virus

Orf virus-NZ2 strain (OrfV) was kindly provided by Dr. Andrew Mercer (University of Otago). OrfV was produced and purified as described previously.[14] Briefly, clarified crude supernatant was subjected to depth filtration, followed by tangential flow filtration, gradient ultracentrifugation, and dialysis. OrfV was titrated by 50% tissue culture infectious dose ($TCID_{50}$) assay on SSF cells as previously described.[14]

In Vivo tumor model and virus therapy

The syngeneic orthotopic ID8 ovarian cancer model was established as previously described.[17,42] Briefly, 1e+06 ID8 cells were injected into the left ovarian bursa of 8-week-old female C57BL/6 mice. At 60 days postimplantation, mice presented with signs similar to advanced-stage EOC, including a large primary tumor, development of ascites in the peritoneal cavity, and secondary lesions on the peritoneum walls and other organs within the peritoneal cavity. ID8 tumor-bearing mice were treated 60 days following tumor implantation with either PBS or OrfV at a dose of 5e+07 PFU administered by intraperitoneal injection. There was no significant difference in weight between the two groups before or after treatment with OrfV or PBS. Endpoints were either at prescribed times postvirus administration or when mice reached the endpoint criteria for survival assessment, including distended abdomen interfering with mobility, hunched fur, irregular breathing, or isolated behavior.

Virus titration

To quantify infectious OrfV in the ascites fluid following treatment, ID8 tumor-bearing mice were treated with 5e+07 PFU OrfV intraperitoneally on day 60. At 24, 48, 72, 96, and 120 h postvirus administration, peritoneal ascites fluid was collected and subjected to 3 freeze-thaw cycles to disrupt cell membranes and release intracellular virus. Clarified virus-containing supernatant was collected after 10 min of centrifugation at 10,000 $\times$ g at 4°C. Virus-containing supernatant was titrated by $TCID_{50}$ on SSFs for OrfV, and $TCID_{50}$ values were converted to PFU by multiplying by 0.69 as previously described.[43,44]

Figure 8. Identification of optimal OrfV re-administration timing using tumor-to-leukocyte ratios

ID8 tumor-bearing mice were treated with PBS or a single dose of 5×10^7 PFU OrfV intraperitoneally on day 60 and subjected to peritoneal lavage fluid sampling for the analysis of individual -tumor-to-leukocyte ratios over time by flow cytometry. (A) The ratio of the total number of leukocytes to secondary tumor lesions in the ascites fluid of mice treated with OrfV or PBS over time. (B) The number of tumor cells in the ascites fluid of OrfV- or PBS-treated mice over time. The ratio of specific immune effector cells to tumor cells within the peritoneal cavity following treatment was quantified over time, including (C) neutrophils, (D) macrophages, (E) NK cells, (F) classical DCs, (G) cDC1 ($CD103^+$s), (H) $CD8^+$ T cells, (I) $CD4^+$ T cells, and (J) B cells. All of the graphs are expressed in a logarithmic scale, with arrows highlighting the wane of individual leukocyte responses. Following intraperitoneal administration of PBS or OrfV on day 60, ID8 mice received a second dose of PBS or 5e+07 PFU OrfV either on (K and L) day 65, (K and M) day 70, or (K and N) day 75, and survival was monitored. The survival between groups was compared by Mantel-Cox log rank test. Significance was determined by $p \leq 0.05$ (*$p \leq 0.05$; **$p \leq 0.01$). Graphs show mean and SE.

Flow cytometry analysis of immune responses

Mice were anesthetized on days 1, 3, 5, 8, 10, 15, and 21 following OrfV or PBS administration, and peripheral blood or peritoneal lavage fluid was collected for immune cell phenotyping by flow cytometry. Retro-orbital blood and peritoneal lavage fluid samples were collected into heparinized microtubes to prevent clotting and then were kept on ice during transport and processing. Peritoneal lavages were conducted under isoflurane anesthesia as follows. First, 3 mL of room temperature PBS was injected into the abdominal cavity, followed by 10 s of gentle massage to distribute the PBS. Second, ?1.5 mL of peritoneal fluid was withdrawn, and Vetbond Tissue Adhesive (Santa Cruz Animal Health, catalog no. sc-361931) was immediately applied to prevent leakage. Volumes were recorded to facilitate normalizing flow cytometry data on a per-microliter basis, and erythrocytes were lysed using ammonium-chloride-potassium lysing buffer. Leukocytes were suspended in RPMI 1640 media (HyClone, catalog no. SH3002701) containing 10% HI FBS and 0.01% β-metacaptoethanol. When cytokine expression was analyzed, samples were incubated for 1 h before the addition of brefeldin A (eBiosciences, catalog no. 00-4506-51). Samples were then incubated for an additional 4 h before staining. All of the reagents listed below are from Biolegend, unless otherwise specified. Isolated cells had Fc receptors blocked (anti-CD16/32, catalog no. 101320) for 15 min at 4°C. Samples were divided and stained across several panels with surface markers for the identification of discrete immune cell populations for 20 min at 4°C in the dark, as outlined in Tables S1 and S2. Cytokine secretion was examined following treatment with fixation buffer (catalog no. 420801) and permeabilization buffer (catalog no. 421002). Cells were washed and suspended in 200 μL fluorescence-activated cell sorting (FACS) buffer for flow cytometry analysis. Tumor-specific CTL responses were quantified as described previously.[45] Flow cytometry samples were run using the FACS Canto II (BD Biosciences) and analyzed using FlowJo software version 10 (FlowJo LLC). An example of the gating strategy that was used is shown in Figure S1.

Tumor-directed antibody responses

Tumor-associated and OrfV-directed antibody responses were quantified by flow cytometry on HI plasma and cell-free ascites fluid collected from untreated tumor-free naive or ID8 tumor-bearing mice 10 and 21 days following treatment with PBS or OrfV as previously described.[46,47] Tumor-associated and OrfV-directed antibody binding was represented as a function of positive anti-mouse IgG1-Alexa Fluor 488 (BioLegend, catalog no. 406625) secondary antibody signals from target ID8 or OrfV-infected Vero cells. Antibody data were analyzed by first subtracting the background fluorescence of control wells from each sample. Then, curves were generated from the mean fluorescence intensity of the positive secondary antibody signal from target cells treated with serial dilutions of samples. The area under the curve was then calculated for each sample and graphed alongside tumor-free and tumor-bearing untreated animal controls.

Quantification of cytokines

Peritoneal lavage fluid was harvested from ID8 tumor-bearing mice on days 1, 3, 5, 8, 10, 15, and 21 following intraperitoneal treatment with OrfV or PBS. Lavage fluid was centrifuged at 500 × *g* for 5 min to facilitate the collection of cell-free clarified ascites fluid, which was diluted 1:2 before the assay. Cytokines present within all of the samples were analyzed using the LEGENDplex mouse Anti-Viral Response Panel (BioLegend, catalog no. 740622) performed according to the manufacturer's protocol. Cytokines within samples were measured on a FACS Canto flow cytometer (BD Biosciences). The observed concentrations for each cytokine on a per sample basis were calculated using the LEGENDplex Data Analysis Software (BioLegend). Cytokine concentrations were expressed in nanograms per milliliter.

Virus neutralization assay

The viral neutralizing effect of OrfV-directed antibodies within plasma and cell-free ascites fluid of untreated naive or ID8 mice treated with OrfV or PBS was assessed by viral neutralizing assay. OrfV at an MOI of 3 was incubated with serial dilutions of HI OrfV-directed antibody-containing plasma and cell-free ascites fluid samples for 1 h at 37°C and 5% CO_2, followed by incubation with 1e+04 ID8 target cells for 48 h. Virus-induced cytopathic and cytolytic effects on ID8 cells were visualized by light microscopy at 20× magnification. Viral oncolysis of ID8 cells was performed by a resazurin metabolic activity assay, and 1e+04 ID8 cells were seeded into 96-well plates. OrfV at an MOI of 3 was incubated with serial dilutions of OrfV-directed antibody-containing plasma and cell-free ascites fluid samples for 1 h at 37°C and 5% CO_2 before the infection of the plated ID8 cells. After 48 h, 0.5 mg/mL resazurin sodium salt (Sigma-Aldrich) was added for 2 h before data acquisition by the fluorescent plate reader (excitation wavelength: 535/25 nm, emission wavelength: 590/35 nm). Relative metabolic activity was calculated by dividing the fluorescent output of treatment cells by untreated control cells.

Statistical analyses

GraphPad Prism version 9 for Mac (GraphPad) was used for all of the graphing and statistical analyses. Survival curves were plotted using the Kaplan-Meier method, and differences between groups were queried using the log rank Mantel-Cox test. Immune response data, which involved one variable, were assessed by one-way ANOVA with Tukey's multiple comparisons test, or by the Student's two-tailed t test. Data that involved two variables were assessed by two-way ANOVA with Tukey's multiple comparisons test. All of the reported p values were two sided and were considered significant at $p \leq 0.05$. Graphs show means and standard error (SE)s.

DATA AND CODE AVAILABILITY

Data are available upon reasonable request.

SUPPLEMENTAL INFORMATION

Supplemental information can be found online at https://doi.org/10.1016/j.omto.2023.100748.

ACKNOWLEDGMENTS

Funding was provided by a Discovery Grant from the Natural Sciences and Engineering Research Council of Canada to S.K.W. (no.

499834), and operating grants from the Cancer Research Society (to S.K.W., nos. 843296 and 946085). Stipend funding was obtained from the Ontario Graduate Scholarship, the Nora Cebotarev Memorial Graduate Scholarship, and the Ellen Nilsen Memorial Scholarship (to J.A.M.); and the Canadian Graduate Scholarship-Doctoral Award, the Ontario Graduate Scholarship, and the Ontario Veterinary College Graduate Scholarship (to J.P.v.V.). We thank Campus Animal Facilities at the University of Guelph for animal care services.

AUTHOR CONTRIBUTIONS

Conception and design: J.P.v.V. and S.K.W.; development of the methodology: J.A.M., J.P.v.V., and S.K.W.; acquisition of the data: J.A.M., J.P.v.V., J.G.E.Y., L.A.S., K.M., M.M.G., M.P., and J.J.P; analysis and interpretation of the data: J.A.M., J.P.v.V., and S.K.W.; writing, review, and/or revision of the manuscript: J.A.M., J.P.v.V., J.G.E.Y., A.V.-P, G.A.W., K.K., B.W.B., and S.K.W.

DECLARATION OF INTERESTS

B.W.B. is the chief operating officer of ImmunoCeutica, which is dedicated to the research and development of immunoceuticals. S.K.W. is a scientific founder of Avamab Pharma, a preclinical, prerevenue stage company dedicated to the research and development of adeno-associated virus (AAV) gene therapies for the treatment and prevention of infectious diseases. S.K.W. is a cofounder and the chief scientific officer of Inspire Biotherapeutics, a preclinical, prerevenue stage gene therapy company developing AAV–based therapies for monogenic lung diseases. She is also an inventor on issued patents in Canada and the United States for the AAV6.2FF capsid, which is owned by the University of Guelph and licensed to Avamab Pharma, Inspire Biotherapeutics, and Cellastra. From 2020 to February 2023, she was an unpaid scientific advisor for Cellastra, which is dedicated to the research and development of gene therapies targeting the root causes of scarring. She is a co-inventor on a pending US and Canadian patent for the engineered Newcastle disease virus vector and uses thereof, filed/owned by the University of Guelph.

REFERENCES

1. Society, A.C. (2022). Key Statistics for Ovarian Cancer. https://www.cancer.org/cancer/ovarian-cancer/about/key-statistics.html.
2. Matulonis, U.A., Sood, A.K., Fallowfield, L., Howitt, B.E., Sehouli, J., and Karlan, B.Y. (2016). Ovarian cancer. Nat. Rev. Dis. Primers *2*, 16061.
3. Bowtell, D.D., Böhm, S., Ahmed, A.A., Aspuria, P.J., Bast, R.C., Beral, V., Berek, J.S., Birrer, M.J., Blagden, S., Bookman, M.A., et al. (2015). Rethinking ovarian cancer II: reducing mortality from high-grade serous ovarian cancer. Nat. Rev. Cancer *15*, 668–679.
4. Ma, X.Y., Hill, B.D., Hoang, T., and Wen, F. (2022). Virus-inspired strategies for cancer therapy. Semin. Cancer Biol. *86*, 1143–1157.
5. Wang, L., Chard Dunmall, L.S., Cheng, Z., and Wang, Y. (2022). Remodeling the tumor microenvironment by oncolytic viruses: beyond oncolysis of tumor cells for cancer treatment. J. Immunother. Cancer *10*, e004167.
6. Gujar, S., Pol, J.G., and Kroemer, G. (2018). Heating it up: Oncolytic viruses make tumors 'hot' and suitable for checkpoint blockade immunotherapies. Oncoimmunology *7*, e1442169.
7. Prestwich, R.J., Ilett, E.J., Errington, F., Diaz, R.M., Steele, L.P., Kottke, T., Thompson, J., Galivo, F., Harrington, K.J., Pandha, H.S., et al. (2009). Immune-mediated antitumor activity of reovirus is required for therapy and is independent of direct viral oncolysis and replication. Clin. Cancer Res. *15*, 4374–4381.
8. Galivo, F., Diaz, R.M., Wongthida, P., Thompson, J., Kottke, T., Barber, G., Melcher, A., and Vile, R. (2010). Single-cycle viral gene expression, rather than progressive replication and oncolysis, is required for VSV therapy of B16 melanoma. Gene Ther. *17*, 158–170.
9. Wang, R., Wang, Y., Liu, F., and Luo, S. (2019). Orf virus: A promising new therapeutic agent. Rev. Med. Virol. *29*, e2013.
10. Friebe, A., Friederichs, S., Scholz, K., Janssen, U., Scholz, C., Schlapp, T., Mercer, A., Siegling, A., Volk, H.D., and Weber, O. (2011). Characterization of immunostimulatory components of orf virus (parapoxvirus ovis). J. Gen. Virol. *92*, 1571–1584.
11. Haig, D.M., and Fleming, S. (1999). Immunomodulation by virulence proteins of the parapoxvirus orf virus. Vet. Immunol. Immunopathol. *72*, 81–86.
12. Haig, D.M., and Mercer, A.A. (1998). Ovine diseases. Orf. Vet. Res. *29*, 311–326.
13. Bukar, A.M., Jesse, F.F.A., Abdullah, C.A.C., Noordin, M.M., Lawan, Z., Mangga, H.K., Balakrishnan, K.N., and Azmi, M.M. (2021). Immunomodulatory Strategies for Parapoxvirus: Current Status and Future Approaches for the Development of Vaccines against Orf Virus Infection. Vaccines (Basel) *9*, 1341.
14. van Vloten, J.P., Minott, J.A., McAusland, T.M., Ingrao, J.C., Santry, L.A., McFadden, G., Petrik, J.J., Bridle, B.W., and Wootton, S.K. (2021). Production and purification of high-titer OrfV for preclinical studies in vaccinology and cancer therapy. Mol. Ther. Methods Clin. Dev. *23*, 434–447.
15. Rintoul, J.L., Lemay, C.G., Tai, L.H., Stanford, M.M., Falls, T.J., de Souza, C.T., Bridle, B.W., Daneshmand, M., Ohashi, P.S., Wan, Y., et al. (2012). ORFV: a novel oncolytic and immune stimulating parapoxvirus therapeutic. Mol. Ther. *20*, 1148–1157.
16. Deng, H., Xiao, B., Huang, Y., Weng, K., Chen, J., Li, K., Wu, H., Luo, S., and Hao, W. (2022). The Combined Use of Orf Virus and PAK4 Inhibitor Exerts Anti-tumor Effect in Breast Cancer. Front Microbiol. *13*, 845259.
17. van Vloten, J.P., Matuszewska, K., Minow, M.A.A., Minott, J.A., Santry, L.A., Pereira, M., Stegelmeier, A.A., McAusland, T.M., Klafuric, E.M., Karimi, K., et al. (2022). Oncolytic Orf virus licenses NK cells via cDC1 to activate innate and adaptive antitumor mechanisms and extends survival in a murine model of late-stage ovarian cancer. J. Immunother. Cancer *10*, e004335.
18. Roberts, E.W., Broz, M.L., Binnewies, M., Headley, M.B., Nelson, A.E., Wolf, D.M., Kaisho, T., Bogunovic, D., Bhardwaj, N., and Krummel, M.F. (2016). Critical Role for CD103(+)/CD141(+) Dendritic Cells Bearing CCR7 for Tumor Antigen Trafficking and Priming of T Cell Immunity in Melanoma. Cancer Cell *30*, 324–336.
19. Costantini, C., Calzetti, F., Perbellini, O., Micheletti, A., Scarponi, C., Lonardi, S., Pelletier, M., Schakel, K., Pizzolo, G., Facchetti, F., et al. (2011). Human neutrophils interact with both 6-sulfo LacNAc+ DC and NK cells to amplify NK-derived IFN {gamma}: role of CD18, ICAM-1, and ICAM-3. Blood *117*, 1677–1686.
20. Sun, R., Luo, J., Li, D., Shu, Y., Luo, C., Wang, S.S., Qin, J., Zhang, G.M., and Feng, Z.H. (2014). Neutrophils with protumor potential could efficiently suppress tumor growth after cytokine priming and in presence of normal NK cells. Oncotarget *5*, 12621–12634.
21. Böttcher, J.P., Bonavita, E., Chakravarty, P., Blees, H., Cabeza-Cabrerizo, M., Sammicheli, S., Rogers, N.C., Sahai, E., Zelenay, S., and Reis e Sousa, C. (2018). NK Cells Stimulate Recruitment of cDC1 into the Tumor Microenvironment Promoting Cancer Immune Control. Cell *172*, 1022–1037.e14.
22. Boudreau, J.E., Stephenson, K.B., Wang, F., Ashkar, A.A., Mossman, K.L., Lenz, L.L., Rosenthal, K.L., Bramson, J.L., Lichty, B.D., and Wan, Y. (2011). IL-15 and type I interferon are required for activation of tumoricidal NK cells by virus-infected dendritic cells. Cancer Res. *71*, 2497–2506.
23. Berraondo, P., Sanmamed, M.F., Ochoa, M.C., Etxeberria, I., Aznar, M.A., Pérez-Gracia, J.L., Rodríguez-Ruiz, M.E., Ponz-Sarvise, M., Castañón, E., and Melero, I. (2019). Cytokines in clinical cancer immunotherapy. Br. J. Cancer *120*, 6–15.
24. Russell, S.J., and Barber, G.N. (2018). Oncolytic Viruses as Antigen-Agnostic Cancer Vaccines. Cancer Cell *33*, 599–605.
25. Costantini, C., and Cassatella, M.A. (2011). The defensive alliance between neutrophils and NK cells as a novel arm of innate immunity. J. Leukoc. Biol. *89*, 221–233.

26. Lei, X., Lei, Y., Li, J.K., Du, W.X., Li, R.G., Yang, J., Li, J., Li, F., and Tan, H.B. (2020). Immune cells within the tumor microenvironment: Biological functions and roles in cancer immunotherapy. Cancer Lett. *470*, 126–133.

27. Guillerey, C., Huntington, N.D., and Smyth, M.J. (2016). Targeting natural killer cells in cancer immunotherapy. Nat. Immunol. *17*, 1025–1036.

28. Friebe, A., Siegling, A., Friederichs, S., Volk, H.D., and Weber, O. (2004). Immunomodulatory effects of inactivated parapoxvirus ovis (ORF virus) on human peripheral immune cells: induction of cytokine secretion in monocytes and Th1-like cells. J. Virol. *78*, 9400–9411.

29. Li, J., O'Malley, M., Urban, J., Sampath, P., Guo, Z.S., Kalinski, P., Thorne, S.H., and Bartlett, D.L. (2011). Chemokine expression from oncolytic vaccinia virus enhances vaccine therapies of cancer. Mol. Ther. *19*, 650–657.

30. Parker, J.N., Meleth, S., Hughes, K.B., Gillespie, G.Y., Whitley, R.J., and Markert, J.M. (2005). Enhanced inhibition of syngeneic murine tumors by combinatorial therapy with genetically engineered HSV-1 expressing CCL2 and IL-12. Cancer Gene Ther. *12*, 359–368.

31. Nguyen, H.M., Guz-Montgomery, K., and Saha, D. (2020). Oncolytic Virus Encoding a Master Pro-Inflammatory Cytokine Interleukin 12 in Cancer Immunotherapy. Cells *9*, 400.

32. Pearl, T.M., Markert, J.M., Cassady, K.A., and Ghonime, M.G. (2019). Oncolytic Virus-Based Cytokine Expression to Improve Immune Activity in Brain and Solid Tumors. Mol. Ther. Oncolytics *13*, 14–21.

33. Granot, Z., and Jablonska, J. (2015). Distinct Functions of Neutrophil in Cancer and Its Regulation. Mediators Inflamm. *2015*, 701067.

34. Aras, S., and Zaidi, M.R. (2017). TAMeless traitors: macrophages in cancer progression and metastasis. Br. J. Cancer *117*, 1583–1591.

35. Robinson-Smith, T.M., Isaacsohn, I., Mercer, C.A., Zhou, M., Van Rooijen, N., Husseinzadeh, N., McFarland-Mancini, M.M., and Drew, A.F. (2007). Macrophages mediate inflammation-enhanced metastasis of ovarian tumors in mice. Cancer Res. *67*, 5708–5716.

36. Bak, S.P., Walters, J.J., Takeya, M., Conejo-Garcia, J.R., and Berwin, B.L. (2007). Scavenger receptor-A-targeted leukocyte depletion inhibits peritoneal ovarian tumor progression. Cancer Res. *67*, 4783–4789.

37. Martin, S.D., Brown, S.D., Wick, D.A., Nielsen, J.S., Kroeger, D.R., Twumasi-Boateng, K., Holt, R.A., and Nelson, B.H. (2016). Low Mutation Burden in Ovarian Cancer May Limit the Utility of Neoantigen-Targeted Vaccines. PLoS One *11*, e0155189.

38. Haig, D.M. (2006). Orf virus infection and host immunity. Curr. Opin. Infect Dis. *19*, 127–131.

39. Reguzova, A., Ghosh, M., Müller, M., Rziha, H.J., and Amann, R. (2020). Orf Virus-Based vaccine vector D1701-V induces strong CD8+ T Cell response against the transgene but not against ORFV-Derived epitopes. Vaccines (Basel) *8*, 295.

40. LaRocca, C.J., and Warner, S.G. (2018). Oncolytic viruses and checkpoint inhibitors: combination therapy in clinical trials. Clin. Transl Med. *7*, 35.

41. Buttner, M., and Rziha, H.J. (2002). Parapoxviruses: from the lesion to the viral genome. J. Vet. Med. B Infect Dis. Vet. Public Health *49*, 7–16.

42. Greenaway, J., Henkin, J., Lawler, J., Moorehead, R., and Petrik, J. (2009). ABT-510 induces tumor cell apoptosis and inhibits ovarian tumor growth in an orthotopic, syngeneic model of epithelial ovarian cancer. Mol. Cancer Ther. *8*, 64–74.

43. Bryan, W.R. (1957). Interpretation of host response in quantitative studies on animal viruses. Ann. N. Y Acad. Sci. *69*, 698–728.

44. Wulff, N.H., Tzatzaris, M., and Young, P.J. (2012). Monte Carlo simulation of the Spearman-Kaerber TCID50. J. Clin. Bioinforma *2*, 5.

45. van Vloten, J.P., Santry, L.A., McAusland, T.M., Karimi, K., McFadden, G., Petrik, J.J., Wootton, S.K., and Bridle, B.W. (2019). Quantifying Antigen-Specific T Cell Responses When Using Antigen-Agnostic Immunotherapies. Mol. Ther. Methods Clin. Dev. *13*, 154–166.

46. van Vloten, J.P., Klafuric, E.M., Karimi, K., McFadden, G., Petrik, J.J., Wootton, S.K., and Bridle, B.W. (2019). Quantifying Antibody Responses Induced by Antigen-Agnostic Immunotherapies. Mol. Ther. Methods Clin. Dev. *14*, 189–196.

47. Minott, J.A., van Vloten, J.P., Yates, J.G.E., Chan, L., Wood, G.A., Viloria-Petit, A.M., Karimi, K., Petrik, J.J., Wootton, S.K., and Bridle, B.W. (2022). Multiplex flow cytometry-based assay for quantifying tumor- and virus-associated antibodies induced by immunotherapies. Front Immunol. *13*, 1038340.

OMTO, Volume *31*

Supplemental information

Kinetic analysis of oncolytic OrfV-induced innate and adaptive immune responses in a murine model of late-stage ovarian cancer

Jessica A. Minott, Jacob P. van Vloten, Jake G.E. Yates, Lisa A. Santry, Kathy Matuszewska, Madison Pereira, Melanie M. Goens, Alicia M. Viloria-Petit, Geoffrey A. Wood, Khalil Karimi, James J. Petrik, Byram W. Bridle, and Sarah K. Wootton

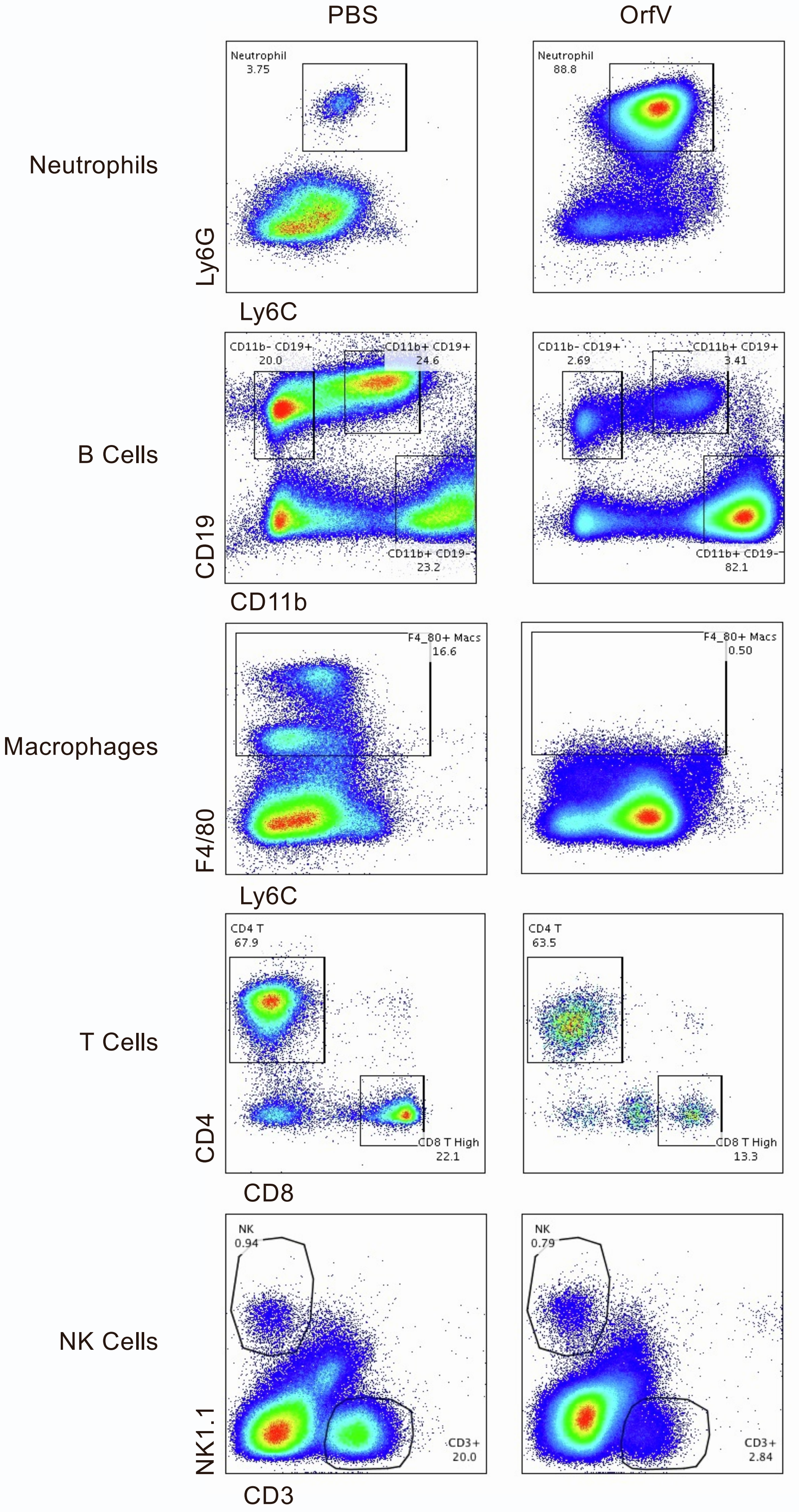

Figure S1: Example of gating used for flow cytometry

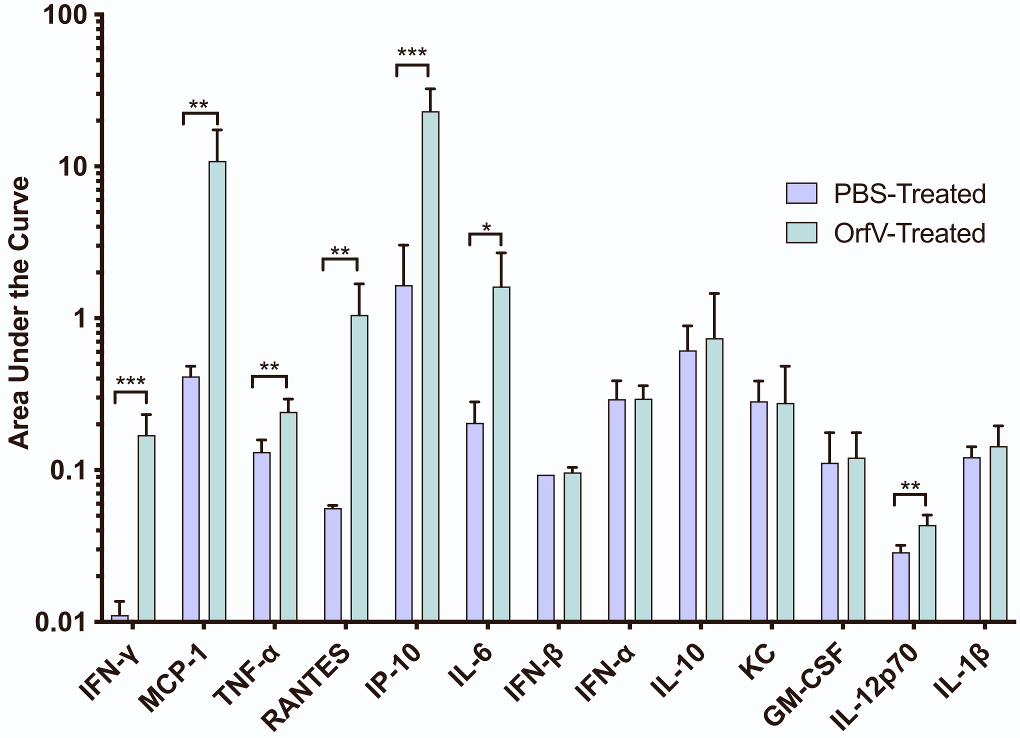

Figure S2. Comparison of the area under the curve for cytokine expression levels in the ascites fluid harvested from OrfV and PBS treated mice and evaluated on days 1, 3, 5, 8, 10, 15, and 21 days post-treatment. Areas under the curve were generated for each individual cytokine and graphically depicted to compare cytokine levels within the peritoneal lavage fluid of OrfV- and PBS-treated mice. P values represent comparison of area under the curve for individual cytokines from PBS or OrfV treated mice using a two-way ANOVA multiple comparisons test. *P<0.05, **P<0.01, ***P<0.001.

Table S1. Markers used for flow cytometry staining to identify specific immunological cell subsets. CD, cluster of differentiation; IFNγ, interferon gamma; MHC- II, major histocompatibility complex molecule II; PD-1, programmed cell death protein 1; PD-L1, Programmed death-ligand 1; TNFα, tumor necrosis factor alpha.

**Leukocyte Subsets**	**Phenotypic Markers**
Neutrophils	CD45.2, Ly6G, Ly6C, CD11b
Macrophages	F4/80, Ly6C, CD45.2, CD11b
NK cells	CD69, NK1.1, CD3ε, CD45.2, CD4, PD-1, PD-L1, CD8α
DCs	CD11b, CD11c, MHC-II, CD8α, CD103, CD40, CD80
cDC1s	CD103, CD11b, CD11c, MHC II, CD8a, CD40, CD80
B cells	CD19, CD45.2
T cells	CD3ε, CD8α, CD4, CD45, IFNγ, TNFα, CD69, PD-1, PD-L1

Table S2. Example staining panel. 7AAD, 7-Aminoactinomycin D; CD, cluster of differentiation; DC, dendritic cell; cDC1, type 1 conventional dendritic cell; FVD, fixable viability dye; IFNγ, interferon gamma; MHC- II, major histocompatibility complex molecule II; NK, natural killer cell; PD-1, programmed cell death protein 1; PD-L1, Programmed death-ligand 1; TNFα, tumor necrosis factor alpha.

	**530 FITC**	**585 PE**	**780B-PECy7**	**670LP PerCP5.5**	**660 APC**	**780R APCCy7**	**450 BV421**	**510 BV510**
Neutrophils, Macrophages and B cells	F4/80 (eBiosciences, Cat#11-4801-85)	Ly6G (BDPharm, Cat#551461)	CD45.2 (BDbio, Cat#560696)	7AAD (Cat#420404)	CD19 (Cat# 152410)	Ly6C (BDbio, Cat#560596)	CD11b (eBioscience, Cat#48-0112-82)	
NK cells	CD69 (Cat#104506)	PD-L1 (Cat# 124308)	CD4 (eBiosciences, Cat#25-0042-82)	PD-1 (Cat#109120)	NK1.1 (Cat#108710)	FVD NIR (Cat#423106)	CD3ε (Cat#100336)	CD8α (Cat#100752)
DCs and cDC1s	CD103	CD80 (eBiosciences, Cat#12-0801-85)	CD11c (Cat#117318)	7AAD	CD40 (eBiosciences, Cat#17-0401-82)	MHC II (eBioscience, Cat#17-5321-82)	CD11b	CD8α
T cells	CD69	TNF-α (eBioscience, Cat#12-7321-82)	CD4	PD-1	IFN-γ (Cat. #505808)	FVD NIR	CD3ε	CD8α
